# Ruddlesden–Popper Hybrid Lead Bromide Perovskite Nanosheets of Phase Pure *n*=2: Stabilized Colloids Stored in the Solid State

**DOI:** 10.1002/anie.202113451

**Published:** 2021-11-10

**Authors:** Rita B. Cevallos‐Toledo, Ignacio Rosa‐Pardo, Raul Arenal, Víctor Oestreicher, Michael Fickert, Gonzalo Abellán, Raquel E. Galian, Julia Pérez‐Prieto

**Affiliations:** ^1^ Institute of Molecular Science University of Valencia c/ Catedrático José Beltrán 2 Paterna Spain; ^2^ Laboratorio de Microscopias Avanzadas (LMA) U. Zaragoza Mariano Esquillor s/n 50018 Zaragoza Spain; ^3^ Instituto de Nanociencia y Materiales de Aragon (INMA) CSIC-U. de Zaragoza Calle Pedro Cerbuna 12 50009 Zaragoza Spain; ^4^ ARAID Foundation 50018 Zaragoza Spain; ^5^ Department of Chemistry and Pharmacy, Chair of Organic Chemistry II and Joint Institute of Advanced Materials and Processes (ZMP) Friedrich-Alexander-Universität Erlangen-Nürnberg (FAU) Nikolaus-Fiebiger Strasse 10 90762 Erlangen Germany; ^6^ Friedrich-Alexander-Universität Erlangen-Nürnberg (FAU) Dr.-Mack Strasse 81 90762 Fürth Germany

**Keywords:** colloids, film, lead halide perovskites, nanodots, nanosheets

## Abstract

Ruddlesden‐Popper lead halide perovskite (RP‐LHP) nano‐nanostructures can be regarded as self‐assembled quantum wells or superlattices of 3D perovskites with an intrinsic quantum well thickness of a single or a few (*n*=2‐4) lead halide layers; the quantum wells are separated by organic layers. They can be scaled down to a single quantum well dimension. Here, the preparation of highly (photo)chemical and colloidal stable hybrid LHP nanosheets (NSs) of ca. 7.4 μm lateral size and 2.5 nm quantum well height (thereby presenting a deep blue emission at ca. 440 nm), is reported for the first time. The NSs are close‐lying and they even interconnect when deposited on a substrate. Their synthesis is based on the use of the *p*‐toluenesulfonic acid/dodecylamine (*p*TS/DDA) ligand pair and their (photo)chemical stability and photoluminescence is enhanced by adding EuBr_2_ nanodots (EuNDs). Strikingly, they can be preserved as a solid and stored for at least one year. The blue emissive colloid can be recovered from the solid as needed by simply dispersing the powder in toluene and then using it to prepare solid films, making them very promising candidates for manufacturing devices.

## Graphical abstract

Quantum confined nanosheets (NSs) of 3D perovskites with a thickness of two lead halide layers can be prepared by using the dodecylammonium/tosylate ligand pair. The addition of Eu‐nanodots to the colloid prevented the NSs from a structural rearrangement and/or coalescence. Deep blue‐emissive colloids were recovered by simply dispersing it in toluene.

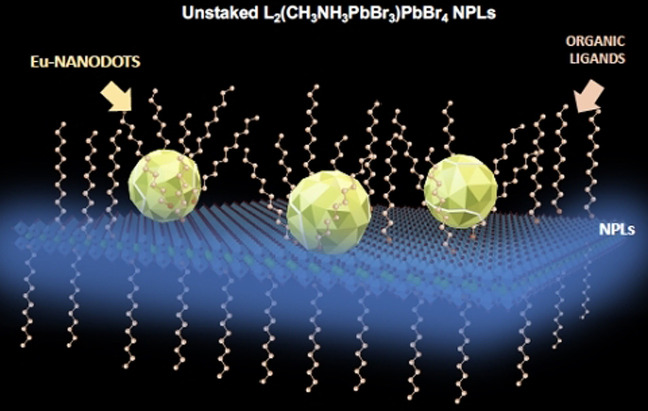

Colloidal lead halide perovskites (LHPs) are relatively new semiconductor materials which are attracting great interest due to their outstanding optoelectronic properties.^[1]^ The most studied materials present the APbX_3_ formula, where A is a small‐sized organic or metal mono‐cation and X is a halide anion and present a three‐dimensional (3D) inorganic framework.^[2]^ LHPs can also be prepared with other stoichiometries and different morphologies.^[3]^ Among them, Ruddlesden‐Popper (RP) lead halide perovskite nanostructures with L_2_[APbX_3_]_*n*−1_PbX_4_ formula, where “L” usually represents a long‐chain alkyl or aromatic ammonium ligand and “*n*” is an integer which stands for the number of lead halide layers held together by weak van der Waals interactions, and sandwiched between organic layers, are cutting‐edge.^[4]^ Maruyama et al. reported the first layered lead halide 2D‐perovskite, specifically (CH_3_(CH_2_)_8_NH_3_)_2_PbI_4_,^[5]^ and soon after Ishihara et al.^[6]^ analyzed the photophysical features of this type of materials.

Ruddlesden‐Popper 2D nanostructures exhibit different features from those of typical 2D perovskites: i) they are integrated by quantum well structures, thereby exhibiting a strong quantum confinement due to their thickness (below the Bohr radius of the material); ii) the quantum confinement occurs without physically thinning the material down to the atomic thickness; and consequently iii) the emission peaks appear at the same wavelength as long as the [A_*n*−1_Pb_*n*_X_3*n*+1_] layer thickness is homogeneous, independently of its lateral dimensions; iv) the excitonic absorption and emission peaks are exceptionally narrow, with a small Stokes shift (<10 meV).

Moreover, the successful manufacture of atomically thin nanosheets with *n=*1 reported by Yang et al. in 2015^[7]^ has shown that the preparation of individually dispersed materials with a single quantum well is feasible.

Perovskite nanosheets with a strong quantum confinement (quantum wells with 1–4 lead halide layers) exhibit a blue‐shifted emission compared to the bulk phase.^[8]^

Among many exciting characteristics of these perovskite nanostructures, the excellent optoelectronic properties, such as their large absorption coefficient, high carrier mobility and long diffusion length, make them promising materials for energy‐related devices, including photodetectors, light‐emitting diodes (LEDs), and solar cells.^[9]^ Namely, LHP nanostructures with a large lateral size, such as NSs, would be easy to incorporate into optical or electronic devices; furthermore, they could be used to improve the moisture tolerance of 3D materials.[^10^, ^11^] However, they present poor chemical stability, especially those of a single quantum well dimension, as they tend to evolve to a mixture of quantum wells with different thicknesses, and consequently to a material with a considerably broad emission.[^9a^, ^9b^, ^12^] The preparation of films by assembling quantum wells differing in their thickness has been envisaged as an opportunity to perform nonradiative energy transfer between them and consequently to prepare tailor‐made energy cascade nanostructures of interest in optoelectronics, but the assembly stability needs to be improved.^[13]^

The surface chemistry of LHPs has been extensively studied.^[14]^ The ionic character of perovskites favors electrostatic interactions between the anchoring group and the perovskite surface. A dynamic equilibrium of bound‐unbound ligands plays a key role in their colloidal stability and emissive properties. The sulfonate anion is a good coordinating ligand of the Pb ions at the perovskite surface; in particular, the use of *p*‐didodecylbenzenesulfonate (*p*DBS) as the only coordinating ligand leads to 3D, green‐emissive nanomaterial.^[15]^ Considering the high acidity of sulfonic acids (p*K*_a_=−5.4 for *p*‐toluenesulfonic acid, *p*TS),^[16]^ which should protonate amines, we embarked on the preparation of strongly quantum‐confined colloidal nanosheets (NSs) of 3D perovskites with a thickness of two lead halide layers by using *p*TS and a long alkyl (dodecylamine, DDA) ligand pair (Scheme 1), named LHP@DDA/*p*TS NSs. There are similarities between these NSs, phase *n=*1 2D perovskite structures^[7]^ and those with phase control of RP perovskites prepared in aqueous media.^[17]^

**Scheme 1:**
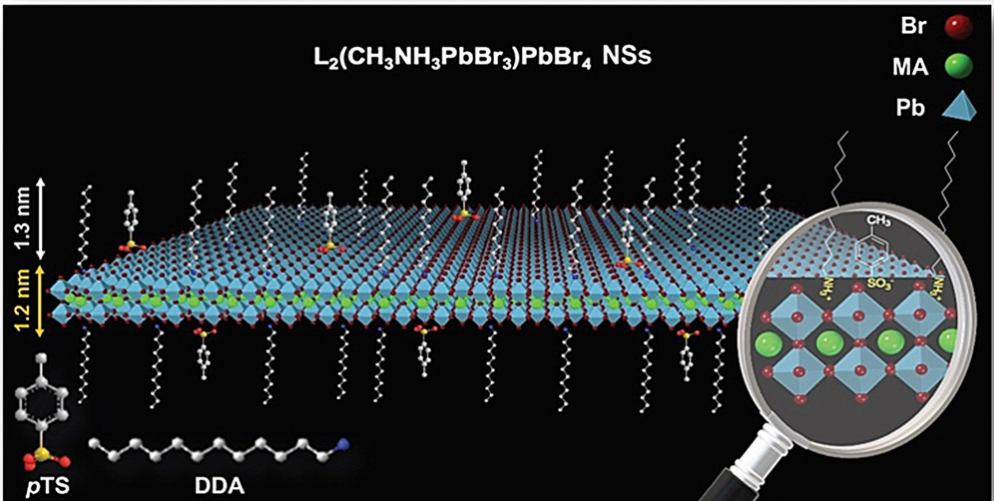
Representative image of LHP@DDA/*p*TS NSs, with a thickness of two lead halide layers, capped with the *p*TS/DDA ligand pair.

The enhanced stabilization of the NSs by adding colloidal EuBr_2_ nanodots (EuNDs of ca. 2.2 nm in diameter) is reported here.

The preparation of LHP@DDA/*p*TS NSs was attempted by means of the ligand‐assisted reprecipitation technique,^[18]^ using a 1:1 methyl ammonium bromide (MABr)/PbBr_2_ molar ratio and DDA and *p*TS as the organic ligands, which were added using two different strategies: i) precursor solution was added to a DDA/*p*TS mixture, and then injected into toluene and ii) precursor solution was added to DDA (solid), and the resulting solution injected into a *p*TS toluene solution (Figure 1 a). Both colloids showed basically two excitonic peaks (at 430 nm and 510 nm) and two emission peaks (at 434 nm and 534 nm); these optical features were consistent with the formation of NSs with a thickness of two lead halide layers and 3D (MAPbBr_3_) perovskite nanoparticles, respectively. However, the second strategy led to a better colloid in terms of dispersibility and purity (formation of only two emissive species), and predominance of the NSs (see Figures S1 and 1b). Different MABr/PbBr_2_ and DDA/*p*TS molar ratios were evaluated, and the data are summarized in Table S1. Briefly, the optimal conditions to eventually obtain the NSs were by using MABr/PbBr_2_, *p*TS/DDA, and MA/DDA molar ratios of 0.8:1.0, 0.7:1.0 and 0.55:1, respectively, and toluene as the solvent (see SI experimental section for more details). Two centrifugation steps (stages I and II in Figure 1 c) were carried out for the purification of the NSs, after which the second supernatant was selected (SN2). It exhibited the expected optical features for the pure NSs (exciton peak at 434 nm, full width half maximum, FWHM=17 nm, and blue emission at 439 nm, FWHM=14 nm; see Figure 1 d). This material was termed LHP@DDA/*p*TS NSs (Scheme 1) and was further characterized by a battery of techniques (see below). Comparatively, the use of DDA as the only organic ligand produced perovskites with a 2D framework (emission peak at 405 nm),^[3b]^ while *p*TS led to the 3D framework material (peak at 536 nm) just as *p*DBS^[15]^ would, see Figure S2.

**Figure 1:**
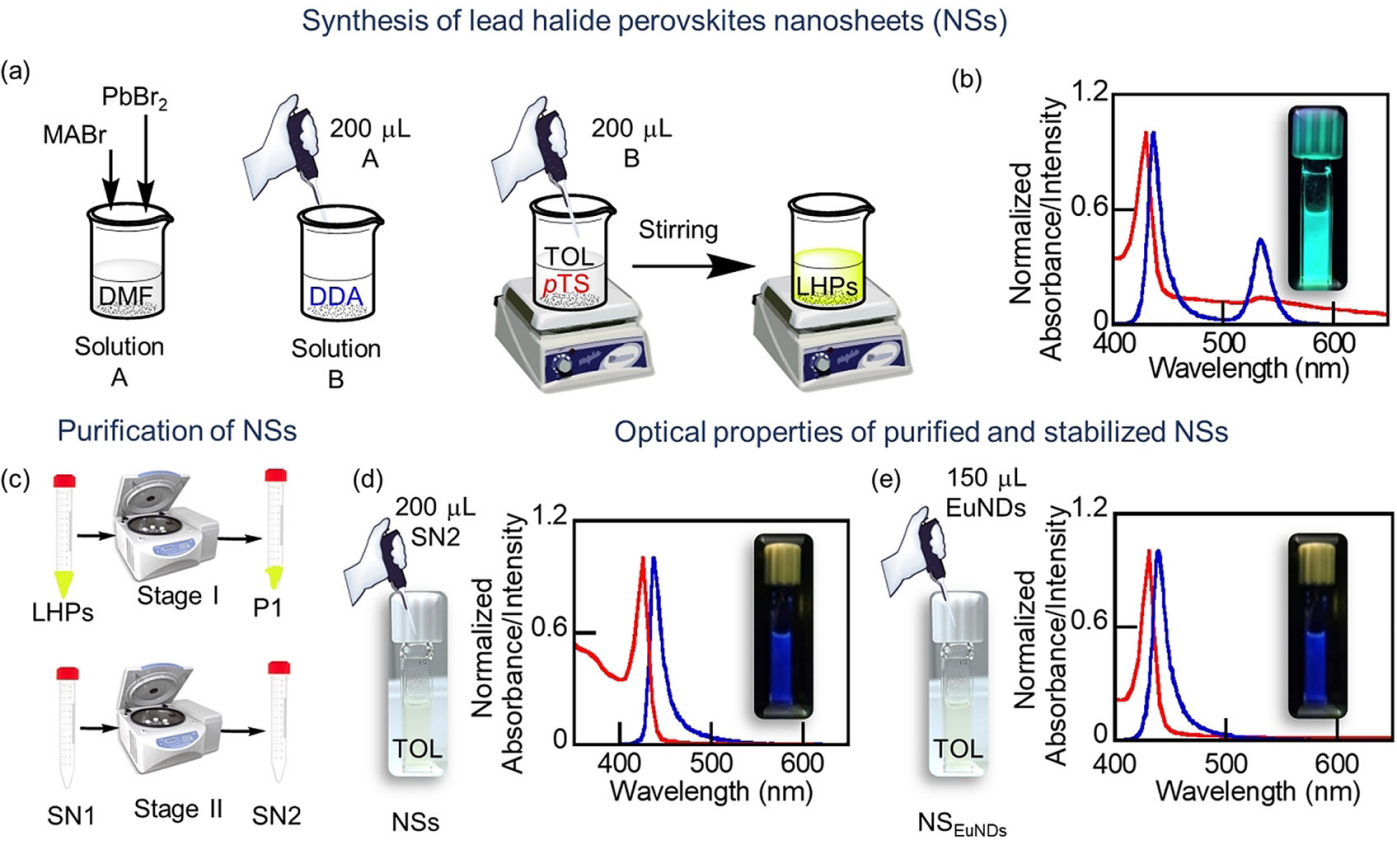
a) Scheme of the LHP@DDA/pTS NSs synthesis prepared by adding a precursor solution A into a DDA (solid) to form a solution B, which it was injected into a toluene solution containing *p*TS; b) normalized absorption and emission spectra (*λ*_ex_=365 nm) of LHPs recorded after the synthesis without any purification step; c) Scheme of the purification of NSs; d),e) normalized absorption and emission spectra (*λ*_ex_=365 nm) of the LHP@DDA/pTS NSs and LHP@DDA/*p*TS_EuNDs_ NSs; inset: photograph of the colloidal dispersion (d) and of the film (e) under UV light.

Although we succeeded in preparing pure, blue emissive NSs, they slowly evolved to other stoichiometries, as shown by the appearance of additional emission peaks at 456 nm and 520 nm, ascribed to NSs with a thickness of three lead halide layers and 3D (MAPbBr_3_) nanoparticles, respectively (see Figure S3a). Therefore, to enhance the chemical stability of the NSs, we used a previously reported strategy but with some modifications.[^19^, ^20^] Specifically, an aliquot of a dispersion of EuNDs (0.02 mg mL^−1^) was added to the as‐synthesized colloidal LHP NSs and the material was separated by centrifugation (see experimental section in SI for further details). The LHP@DDA/*p*TS_EuND_ NSs retained the optical properties of the LHP@DDA/*p*TS NSs (exciton and emission peaks at 431 nm and 439 nm, respectively, Figure 1 e and Figure S4), and increased their chemical stability over time (Figure S3b) as well as under the electron beam of the transmission electron microscope (TEM, compare Figure 2 a and Figure S5). Moreover, the photostability of the colloids under irradiation at 365 nm in air at 700 V using a Xe lamp demonstrated the higher photostability of LHP@DDA/*p*TS_EuND_ (Figure S6). Time‐resolved measurements showed the photoluminescence (PL) lifetimes of LHP@DDA/*p*TS_EuND_ fitted to three components, resulting in PL average lifetimes (*τ*_av_) of 37.7±0.8 ns for LHP@DDA/*p*TS_EuND_ and 39±1 ns for LHP@DDA/*p*TS, thereby indicating the negligible effect of the EuNDs on the PL lifetime of the NSs. However, the PL quantum yield (*Φ*_PL_) of the LHP@DDA/*p*TS_EuND_ NSs nearly doubled that of the LHP@DDA/*p*TS NSs (*Φ*_PL_=20 % and 11 %, respectively). The size and morphology of the LHP@DDA/*p*TS_EuND_ NSs were analyzed by TEM and atomic force microscopy (AFM); see Figure 2. TEM images showed the formation of squared NSs of about 7.4 μm in lateral size (Figure 2 a). High‐angle annular dark‐field scanning TEM (HAADF‐STEM) images (Figure 2 b) and energy dispersive X‐ray spectroscopy (EDS‐STEM) analyses shown in Figure 2 c demonstrated that some EuNDs could be observed at the edge of the NSs. Figures 2 d and S7 show characteristic topographic images obtained by AFM of different LHP@DDA/*p*TS_EuND_ NSs isolated on a SiO_2_/Si substrate. Typically, the width of the NSs was on the μm scale, showing a strong aggregation of different nanosheets. The line profiles displayed terraces with well‐marked steps showing the different step sizes that were present in the sample with a minimum thickness of ca. 2.2–2.5 nm (Figure 2 e). These data are consistent with L_2_[CH_3_NH_3_PbBr_3_]PbBr_4_ NSs formed by two metal lead halide layers (lead halide octahedral size of ca. 0.6 nm)^[21]^ and a ligand layer formed by DDA and *p*TS (ca. 1.3 nm). AFM measurements were performed to analyze the distribution of the different heights; statistics and counts of the step heights accumulated at ca. 2.5 nm, 5 nm, 7.5 nm, 10 nm are shown in the histogram (all heights being multiples of about 2.5 nm); see Figure 2 f.

**Figure 2:**
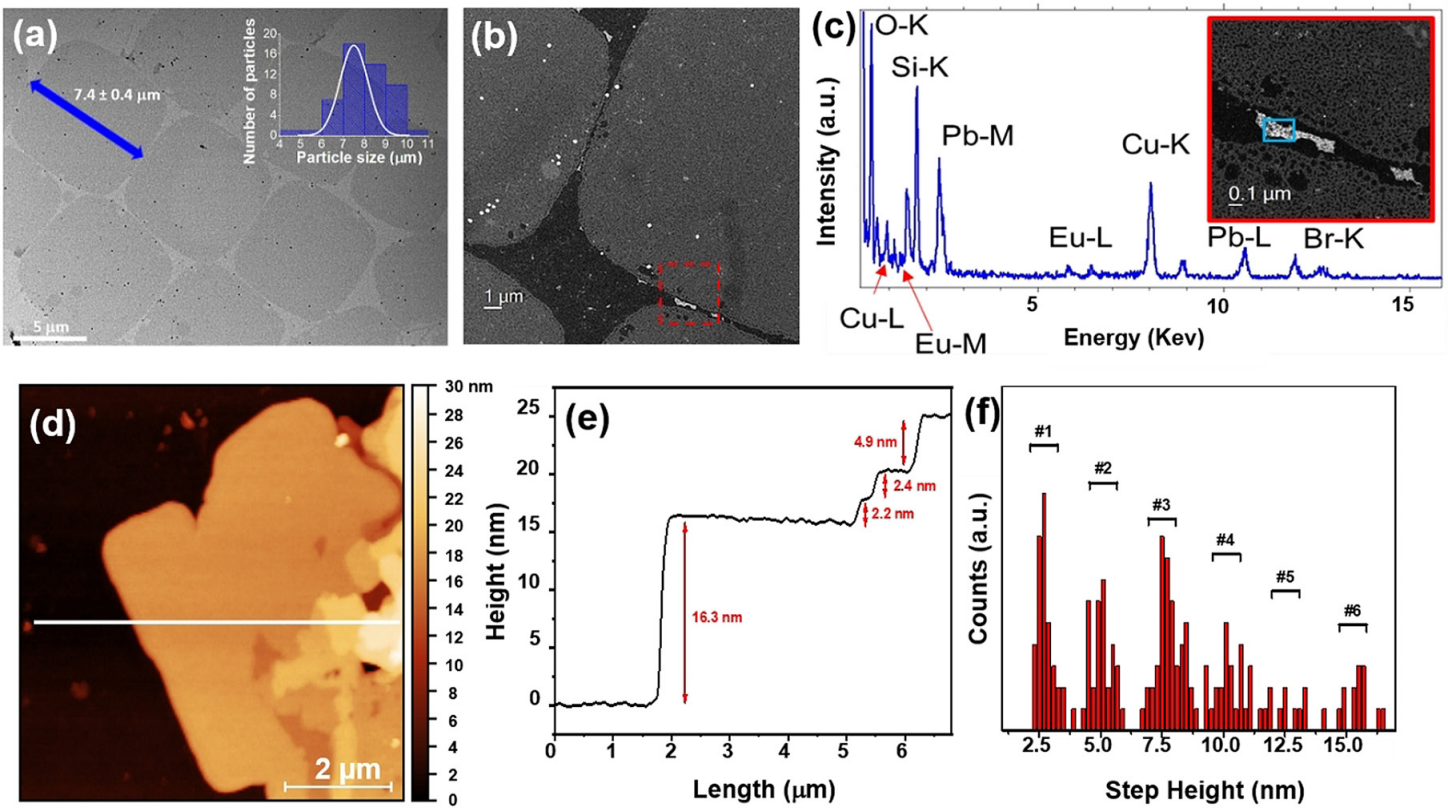
a) TEM image (scale bar 5 μm) and the corresponding histogram. b) HAADF‐STEM image of some NSs; an enlargement can be seen in the red square highlighted in the micrograph, see the inset of the spectrum in (c). c) EDS spectrum collected in the area marked in blue, showing the presence of Eu as well as the other NPL elements. d),e) AFM images of LHP@DDA/*p*TS _EuNDs_ on a SiO_2_/Si substrate and the corresponding line profiles showing the height of the flakes; f) the statistical distribution of the different step heights can be observed in the LHP NSs.

Astonishingly, drying the colloidal LHP@DDA/pTS_EuND_ yielded a yellow powder which remained stable for at least one year; it did not require any special conditions (specifically, it did not need to be stored in darkness or in an inert atmosphere). Moreover, blue‐emissive NSs were recovered as needed by simply dispersing the powder in toluene without adding extra amounts of ligands (Figure 3); therefore, the solid acts as an excellent NS source. A blue‐emissive solid‐state NS film was prepared by depositing the LHP@DDA/*p*TS_EuND_ NS colloid onto a quartz substrate (hydrophobic side) and, surprisingly, it preserved its main optical properties (excitonic and emission peaks at 431 nm and 442 nm, respectively, see Figure 3 and SI for further details).

**Figure 3:**
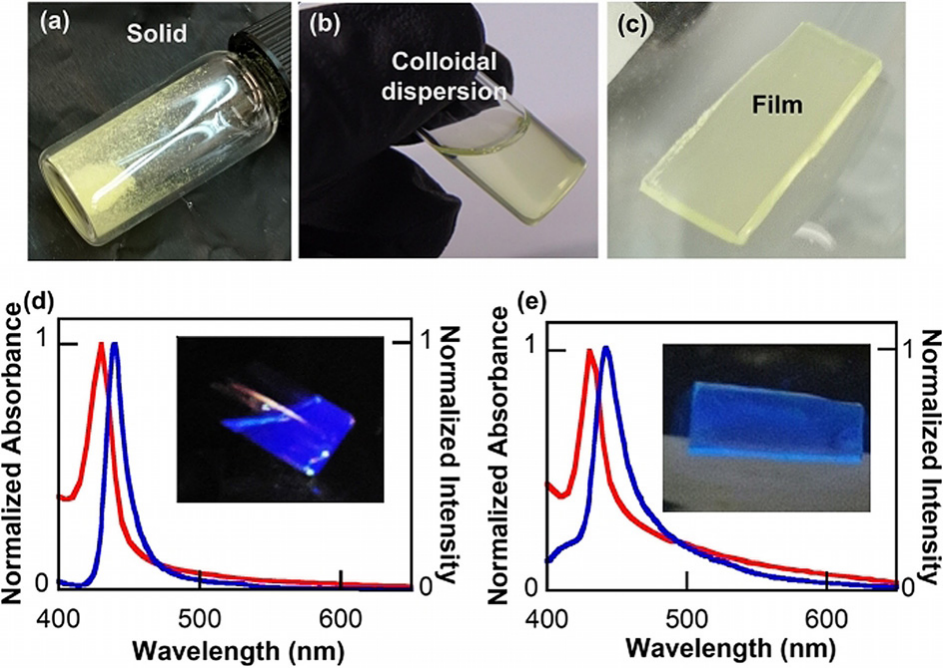
The LHP@DDA/*p*TS_EuND_ solid (a) can be redispersed in toluene to get a colloidal dispersion of the NSs (b), which can be used to prepare a film in a quartz substrate (c) under centrifugal casting. d),e) Normalized absorption and emission spectra of the colloidal dispersion and film of the LHP@DDA/*p*TS_EuND_ NSs.

The powder X‐ray diffraction (XRD) spectra of the LHP@DDA/*p*TS colloid before purification (whose absorption and emission spectra are shown in Figure 1 b) and that of RP‐LHP of phase pure *n=*2 stabilized with EuNDs (LHP@DDA/*p*TS_EuND_) were analyzed in a capillary tube (see further details in the SI). The former showed a set of sharp and intense peaks at low 2‐theta values (see Figure S8a), with reflection expected for the NSs stacked at regular intervals^[22]^ associated with an average spacing between layered NSs of ca. 27 Å, denoted as 00*l*′. Interestingly, this value matched well with the AFM measurements (Figure 2 d–f), suggesting that this is the minimal structural unit constituting the hybrid material and were consistent with the chemical composition of the NSs (Scheme 1). Another set of signals attributable to an average spacing between layered NSs of ca. 32 Å, denoted as 00*l*, were also observed but with very low intensities. These two sets of reflections can be associated with different orientations and/or packing of the organic layers. Similar strong reflections have also been observed in hybrid perovskite nanoplatelets with large lateral sizes prepared in thin films, along with the typical peaks from atomic plane reflections 100 and 200.^[21]^ The additional peaks can be attributed to diffraction planes of the organic ligands and perovskite precursor salts (see Figure S8c for PbBr_2_).^[24]^ In fact, these peaks did not appear in the purified LHP@DDA/*p*TS sample, whose absorption and emission spectra are shown in Figure 1 e and correspond to RP‐LHP phase pure *n*=2. The lack of an exact X‐ray structure for this material impeded the perfect assignment of the peaks. However, the lamellar nature was satisfactorily confirmed.

With respect to LHP@DDA/*p*TS_EuND_, the mixing of the colloidal thin nanosheets with EuNDs followed by drying hindered the stacking and facilitated the observation of the perovskite atomic plane reflections (Figure S8b). Similar observations have been reported by Tisdale et al., who added silica particles to colloidal nanoplatelets before their deposition to form a film.^[21]^

The XRD pattern of both samples showed the typical diffraction peaks, located at 15.03° (100), 21.40° (110), 30.30° (200) and 33.90° (210), which are characteristic of crystalline 3D perovskites, with higher intensity for the *l00* diffraction plane^[21]^ (Figure S8 a,b). Moreover, the two sharp and intense signals centered at ca. 15° and 30° correspond to the distance between two Pb octahedral centers (5.9 Å).^[21]^

The solid was further characterized by X‐Ray photoelectron spectroscopy (XPS), attenuated total reflectance Fourier‐Transform infrared spectroscopy (ATR‐FTIR), thermogravimetric analysis coupled with gas chromatography and mass spectrometry (TG‐GC‐MS), and ^1^H‐NMR (*d*‐DMSO); this information is compiled in the ESI (Figure S9–S24 and Tables S2–S6).

Therefore, the DDA/*p*TS ligand pair brought about the confinement of the perovskite material in two dimension to produce μm‐sized quantum confined nanosheets of 3D perovskites with a thickness of two lead halide layers, while the EuNDs gave them chemical stability. In organic solvents, the perovskite NSs tend to partially evolve into NSs with thicker quantum wells. This can be attributed to a dynamic equilibrium of bound‐unbound ligands on the NS surface in the organic solvent. The EuNDs seem to help in the stabilization of colloidal NSs by stablishing a weak interaction between the EuNDs ligands (with a radial distribution on the nanodot surface) and those of the NSs (with a parallel distribution on the NS surface). Thereby, the EuNDs prevented the NSs from a structural rearrangement and/or coalescence and made the recovery of the colloidal NSs from the solid possible (similar optical properties as the as‐synthesized NSs, see Figures 1 d,e).

To see the specificity of EuNDs in the stabilization of the NSs, semiconductor nanoparticles of CdSe with similar size (2.2 nm) and the same organic ligands to that of the EuNDs (DDA/myristic acid, see experimental section for further details and Figure S25) were also tested. In both cases, addition of the colloid stabilized the nanosheets for at least 8 h (Figure S3c), but the perovskite material was steadily destroyed under prolonged periods in the presence of CdSe. This can be attributed to a weak ligand‐CdSe interaction, thus eventually resulting in perovskite damage.

As mentioned above, the difficulties involved in preparing films from blue‐emitting hybrid perovskite NSs are well known. Their preparation by using anti‐solvent or hot injection procedures results in a low NS concentration, while evaporation and centrifugation procedures, used to increase the concentration, destroy the NS surface, and give rise to agglomeration or staked superstructures.

Remarkably, the long‐term stability in the solid state together with the high processability of the NS colloid herein synthesized enabled the preparation of blue‐emissive solid films, which may be suitable for assembling on a large area on demand and, consequently, be useful for preparing high‐quality films for flexible and ultrathin optoelectronic devices, or they could be combined with other types of 2D materials, through a simple solution process, such as, an ink‐printing method. Research work for this falls outside the scope of this paper but we expect to address it in the near future.

## Conflict of interest

The authors declare no conflict of interest.

## Supporting information

As a service to our authors and readers, this journal provides supporting information supplied by the authors. Such materials are peer reviewed and may be re‐organized for online delivery, but are not copy‐edited or typeset. Technical support issues arising from supporting information (other than missing files) should be addressed to the authors.

Supporting Information
