# Supplementary material for: Ruddlesden–Popper Hybrid Lead Bromide Perovskite Nanosheets of Phase Pure n=2: Stabilized Colloids Stored in the Solid State
Source: Angew Chem Int Ed Engl. 2021 Nov 10;60(52):27312–7. doi: 10.1002/anie.202113451 (PMC9298809; doi:10.1002/anie.202113451)
Supplement: Supplementary file 1 — Supporting Information [file ANIE-60-27312-s001.pdf]

Angewandte Chemie
Eine Zeitschrift der Gesellschaft Deutscher Chemiker

Supporting Information

Ruddlesden–Popper Hybrid Lead Bromide Perovskite Nanosheets of Phase Pure $n = 2$: Stabilized Colloids Stored in the Solid State

Rita B. Cevallos-Toledo, Ignacio Rosa-Pardo, Raul Arenal, Víctor Oestreicher, Michael Fickert, Gonzalo Abellán, Raquel E. Galian, and Julia Pérez-Prieto**

anie_202113451_sm_miscellaneous_information.pdf

Table of Contents

**1. Experimental Procedures**	
- **General information**	**S2**
- **Experimental Section**	**S3**
**2. Results and discussion**	
**Figure S1:** Absorption and emission spectra of the LHP colloid prepared by dissolving DDA and *p*TS in a DMF precursor solution; the resulting mixture was then injected into toluene.<br><br>**Table S1:** Optical properties of LHP colloids using different molar ratios of the precursors.	**S5**
**Figure S2:** Absorption and emission spectra of LHP@DDA (a) and LHP@*p*TS (b) after purification according to Figure S3. Inset: photographs of the dispersion under UV-light irradiation.<br><br>**Figure S3:** Chemical stability over the course of 8 h in air of (a) LHP@DDA/*p*TS NSs, inset: normalized absorption (b) LHP@DDA/*p*TS_{EuND} NSs inset: normalized absorption	**S6**
**Figure S3:** Chemical stability over the course of 8 h in air of (c) LHP@DDA/*p*$TS_{CdSeQDs}$ NSs inset: normalized absorption<br><br>**Figure S4:** Comparison of the normalized emission spectra of LHP@DDA/*p*TS NSs with and without EuNDS<br><br>**Figure S5:** TEM images (scale bar (a) 500 nm and (b) 2 μm) and size distribution of LHP@DDA/pTS NSs (a-c).	**S7**
**Figure S6**: Photostability in air of LHP@DDA/*p*TS and LHP@DDA/*p*TS_{EuND}, $\lambda_{ex} = 365$ nm, 700V. $\lambda_{em} = 439$ nm	**S8**
**Figure S7**: Topographic images obtained by AFM of different LHP@DDA/pTS_{EuND} NSs isolated<br><br>on a SiO2/Si substrate by drop-casting and their corresponding line profiles.	**S9**
**Figure S8:** XRD spectra of LHP@DDA/*p*TS_{EuND} NSs (phase pure n = 2 and mixed phase; black and blue line, respectively).	**S10**
**Figure S9:** XPS spectra of Pb_{4f}, Br_{3d}, O_{1s}, N_{1s}, C_{1s}, and Eu_{3d} of the LHP@DDA/*p*TS_{EuND} NSs	**S11**
**Figure S10:** ATR-FTIR spectra of LHP@DDA/*p*TS_{EuND}, EuNDs, the DDA/*p*TS salt.	**S12**
**Figure S11:** ^{1}H NMR of LHP@DDA/*p*TS_{EuND} in *d*-DMSO.	**S13**
**Figure S12:** ^{1}H-NMR of DDA.<br><br>**Figure S13:** ^{1}H-NMR of *p*TS.	**S14**
**Thermogravimetry Analysis**<br><br>**Figure S14-S24; Table S2-S6.**	**S16-S30**
**Figure S25**: (a) Absorption and emission spectra of CdSe QDs, in toluene (λ_{ex} = 400 nm). (b) Transmission electron microscope (TEM) image of CdSe QDs (scale bar: 200nm). (c) Size distribution of the CdSe QDs.	**S31**

Experimental Procedures

- **General information:**

Reagents. Lead bromide (99.999%, Sigma Aldrich, USA), europium(II) bromide (99.99%, Alfa Aesar, Germany), dodecylamine (98%, Sigma Aldrich, USA), myristic acid (≥ 99%, Sigma Aldrich, USA), N,N-dimethyl-formamide (99.8%, VWR, USA), p-toluene sulfonic acid monohydrate (≥ 98,5%, Sigma Aldrich, USA) and toluene (99.8%, Sigma Aldrich, USA), Trioctylphosphine (TOP, 97%, Sigma Aldrich,USA), selenium powder (100 mesh, 99,99 %, Sigma Aldrich,USA), cadmium acetate dihydrtae (≥ 98 %, Sigma Aldrich,USA), 1-octadecene (ODE; tech. 90 %, Alfa Aesar, Germany) were used in this work.

Characterization.

UV-visible spectroscopy. The UV-Vis spectra were recorded using the UV/VIS/NIR spectrophotometer Lambda 1050, equipped with software PerkinElmer UV Winlab-ink.

Steady state and time resolved fluorescence. Steady-state fluorescence spectra were measured at room temperature using an FLS1000 photoluminiscence spectrometer (Edinburgh Instruments) equipped with a 450 W ozone free xenon arc lamp. The Fluoracle software was used to register the data. Photoluminescence lifetimes were measured at room temperature using the compact fluorescence lifetime spectrometer C11367, Quantaurus-Tau. Fluorescence lifetime software U11487 was used to register and process the data. The photoluminescence decays of colloidal perovskite nanoparticles were fitted with a triexponential function.

Photoluminescence quantum yield measurements. The photoluminescence (PL) quantum yields were recorded at room temperature using a Hamamatsu C9920-02 absolute PL quantum yield measurement system with a mono- chromatic light source (150 W) and integrating sphere. Data were acquired at excitation wavelength of 365 nm, using 1cm×1cm path length quartz cuvettes.

Attenuated total reflectance-Fourier transform infrared spectroscopy. The ATR-FTIR spectra were collected in a Bruker alpha II FTIR spectrometer in the 4000−400 cm^{-1} range in absence of KBr pellets.

Microscopy techniques. Scanning transmission electron microscopy (STEM) was performed on a FEI Titan 80-300kV transmission electron microscope (TEM), working at 300kV. This microscope is equipped with a condenser lens Cs corrector (CETCOR Cs-condenser CEOS Company), a high brightness field emission gun (XFEG) and an Oxford silicon-drift-detector (SDD) for X-ray spectroscopic (EDS) analyses. This technique allows chemical investigations. The atomic force microscopy (AFM) images were obtained using a Bruker Dimension Icon microscope in scan assist mode. The resolution was 512x512 pixel for the statistical images and 1024x1024 for high resolution pictures. All images were taken with a scan rate of 0.4 Hz. Post processing was performed using the Gwydion software. For the statistics, the height differences of the different steps were taken and counted.

Nuclear magnetic resonance (^{1}H-NMR). The spectra were obtained at room temperature in a Bruker DPX300 spectrometer with a 300 MHz Bruker magnet (7 T). The chemical shifts are reported in ppm relative to tetramethylsilane.

Thermogravimetric analysis. TGA coupled to gas-chromatography (GC) and mass spectrometry (MS). Thermogravimetric analysis was carried out on a Netzsch TG 209 F1 Libra instrument. Mass loss and time-dependent temperature profiles in the range of 30 and 900 °C (10 °C/min heating rate) were recorded under a constant flow of He (20 mL/min). The evolved gases detached from the respective samples in combination with the He carrier gas is transferred into the GC system through preheated collector, transfer line, and loop injector a Netzsch TRG 004 at a constant temperature of 300 °C. The gas-chromatographic separation was achieved by an Agilent 8890 GC system equipped with a polysiloxane-coated Elite-5MS capillary column: 30 m length, 0.25 mm diameter, 0.25 µm film thickness. The GC injections fraction of 150 µL were collected at different temperatures according to the steps observed in the TG profile for each sample. Two different conditions were evaluated. In the first case, with the column at 40 °C in order to enhance the separation of the smallest molecules. In this case, after 30 minutes from the last injection the temperature of the column was increased in order to desorb any retained molecule with a 20 °C/min gradient, and finally an isothermal step of 15 min at 280 °C. In the second one, the column was set at 250 °C to characterize the largest molecules. In all the cases, MS measurements were performed on an Agilent 5977B GC/MSD. The obtained data were processed using the Agilent MassHunter Qualitative Analysis10.0 Software and Bibliographic searches where performed with NIST MS Search 2.3.

The X-ray photoelectron spectroscopy (XPS). The XPS spectra were performed in the VG-Microtech Mutilab 3000 equipment, which has a semispherical electron analyzer with 9 channels, pass energy of (2-200 eV) and X-ray radiation source with Mg and Al anodes. Carbon 1s core level peak at 284.5 eV was used as reference.

Powder X-Ray Diffraction (PXRD). Powder X-ray diffraction (PXRD) patterns were obtained employing a PANalytical Empyrean X-ray platform with a capillary platform and copper radiation (Cu Kα = 1.541 78 Å). Measurements were carried out by triplicate in the 2-theta range 4−45° by employing a step size of 0.02 °/step with an integration time of 1 s. The capillary tube used is made of special glass no.0140, with a diameter of 0.05 mm.

- **Experimental Section:**

Synthesis of LHP@DDA/*p*TS NSs. $MAPbBr_3$ hybrid lead halide perovskite was prepared by the ligand assisted reprecipitation technique. A precursor solution A was prepared by mixing $PbBr_2$ (0.037mmol) with MABr (0.029mmol) in 1mL of dimethylformamide, then 200 µL of solution A was added to a vial that contained dodecylamine (DDA, 0.053mmol) to lead to solution B, which was quickly injected into a solution of *p*-toluene sulfonic acid (*p*TS, 0.039 mmol) in toluene (2 mL). The perovskite was immediately formed as a green-yellowish dispersion. The purification of the material was carried out by centrifugation (first at 5,500 rpm for 5 min at 5°C), then the supernatant was centrifugated at 12,500 rpm for 10 min at 5°C and the solid was discarded. The optical properties of the supernatant were recorded using 200 µL of the supernatant dissolved in 2 mL of toluene. It should be noted that all the solvents should be cooled at 8 °C before their use in the synthesis, and the laboratory conditions be temperature ca. 20°C and relative humidity below 60 %.

Synthesis of EuNDs. The synthesis of EuNDs was performed by using the methodology previously described in our group.[1] Briefly, 5 mg of $EuBr_2$ were mixed with 620 µL of dodecylamine (5 mg/mL stock solution in toluene) and 405 µL of myristic acid (5 mg. mL-1 stock solution in toluene) to a final volume in toluene of 2.5 mL. The mixture was sonicated during 2 h in a Branson 2510 Ultrasonic cleaner. They were purified by centrifugation at 5500 rpm, 10 minutes at 5°C, then the precipitate was redispersed in 2.5 mL of toluene.

Preparation of LHP@DDA/*p*TS$_{EuND}$ NSs. LHP@DDA/*p*TS NSs were stabilized by adding a freshly prepared purified EuNDs dispersion (100 µL/mL); specifically, 150 µL of EuNDs dispersion were added to the formed LHP@DDA/*p*TS$_{EuND}$ NSs (reinjection of 200 µL of the second supernatant in 2 mL of toluene). Then the nanosheets were purified by centrifugation at 12,500 rpm for 10 minutes at 5°C. The isolated solid was characterized by a battery of techniques and preserved during at least one year. A small amount of the solid was dispersed in toluene to record the optical properties of the NSs and compare them with those of the freshly synthesized LHP@DDA/*p*TS NSs.

Preparation of LHP@DDA/*p*TS$_{EuND}$ film. 1.8mg of LHP@DDA/*p*TS$_{EuND}$ where redispersed in 1 mL of toluene, then de dispersion was deposited in a quartz hydrophobic side by centrifugation at 12,800 rpm for 4 minutes, then the film was dried in strong and constant flux of nitrogen, its properties were recorded in a FLS1000 photoluminiscence spectrometer (Edinburgh Instruments) equipped with a 450 W ozone free xenon arc lamp, using an integration sphere.

Synthesis of CdSe QDs

a) **Preparation of precursor solutions**

The **Se precursor solution** was prepared by combining 99 mg of Se powder and 5.5 mL of trioctylphosphine (TOP) in a 50 mL round bottom flask. The metallic Se dissolves readily at 45 °C via stirring with a magnetic stir bar. The stirring was kept until the injection.

The **Cd precursor solution** was prepared by mixing 53 mg of cadmium acetate dihydrate, 477 mg of myristic acid (2,09 mmoles), and 5.5 mL of octadecene in a 50 mL round-bottom flask. Then, a magnetic stir bar was added to the flask and the precursor solution was stirred and warmed to 130 °C. The temperature and stirring were kept until the injection.

b) **QDs synthesis**

In a third 50 mL round-bottom flask was prepared the growth solution adding 10 mL of octadecene and 537 mg of dodecylamine (2,90 mmoles). Then, this solution was heated until 165 °C and was immediately injected at the same time 1 ml of **Se and Cd precursor solution**. After 1 min of reaction time, the growth solution was cooled in an ice bath.

c) **QDs purification step**

10 mL of the CdSe QDs solution was transferred into a centrifuge tube. Then, 20 mL of acetone was added into the solution and centrifuged at 4000 rpm for 10 min. The supernatant was discarded, and the yellow pellet was resuspended using 2 mL of toluene.

Results and Discussion

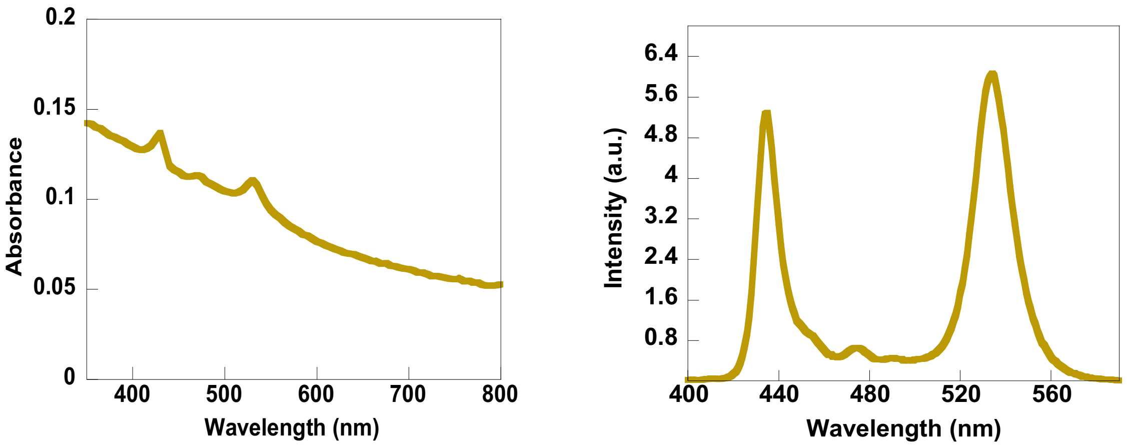

Figure S1. Absorption and emission spectra of the LHP colloid prepared by dissolving DDA and *p*TS in a DMF precursor solution and the resulting mixture was then injected into toluene.

Table S1. Optical properties of LHP colloids under different precursors and ligands molar ratios.

$MABr/PbBr_2$ molar ratio	λ_{max} absorption (nm)	λ_{max} emission (nm)
**0.5:1**	a	a
0.8:1	**433**	**437, 534 (small)**
**1:1**	432	436, 534 (ca. 50 %)

[a] Not observed under this condition.

DDA/*p*TS molar ratio	λ_{max} absorption (nm)	λ_{max} emission (nm)
1:0.7	**429**	**440**
**1:1**	427	437(465, shoulder), 524
**1:1.2**	430	436, 530
**1:1.5**	433	448 (460, shoulder)

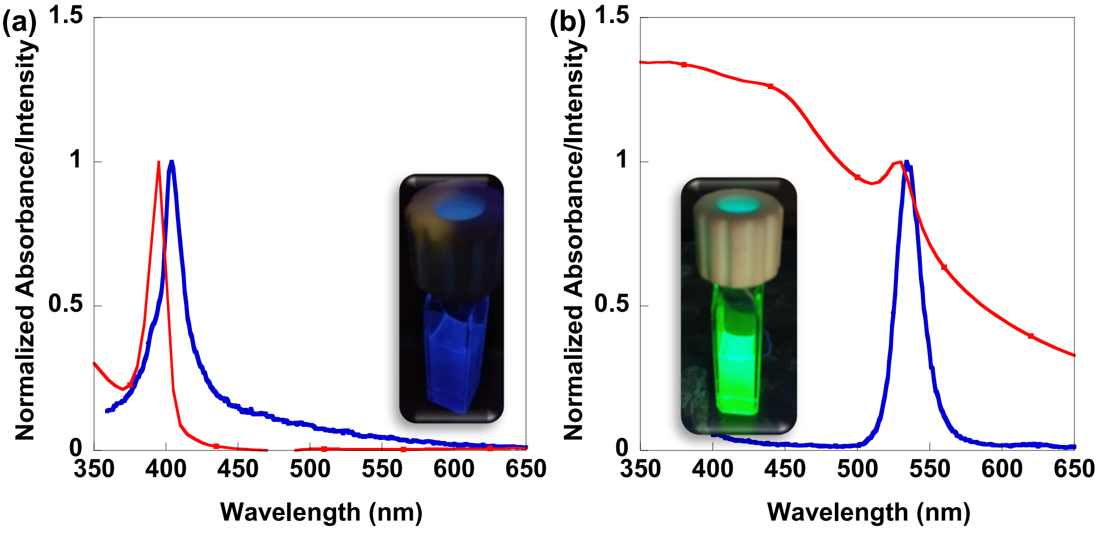

Figure S2. Absorption and emission spectra of LHP@DDA (a) and LHP@*p*TS (b) after purification according to Figure S3. Inset: photographs of the dispersion under UV-light irradiation.

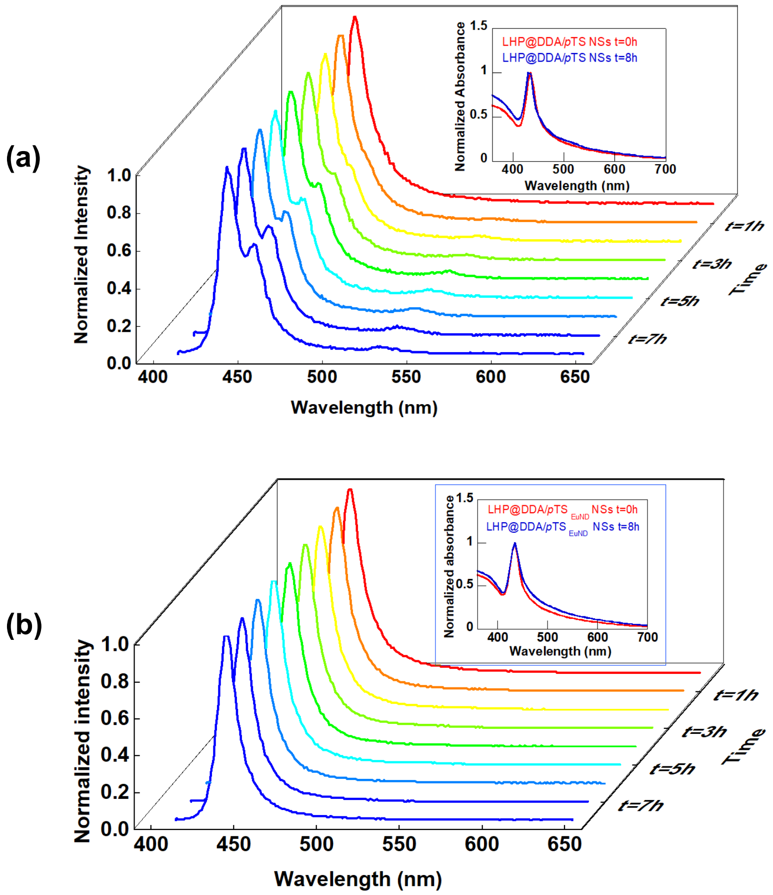

(c)
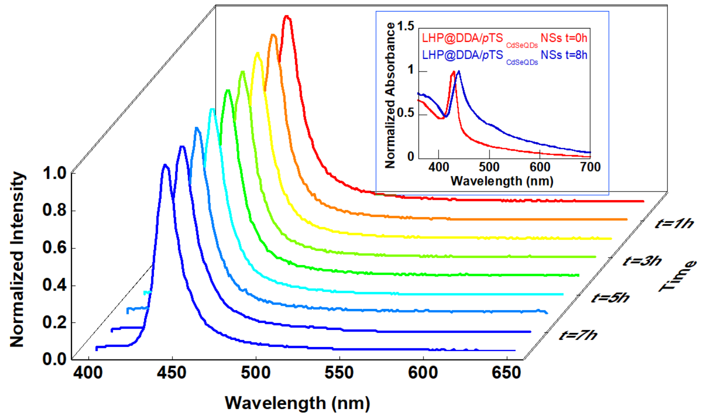

Figure S3. Chemical stability over the course of 8 h in air of (a) LHP@DDA/*p*TS NSs, inset: normalized absorbance (b) LHP@DDA/*p*TS$_{EuND}$ NSs inset: normalized absorbance and (c) LHP@DDA/*p*TS$_{CdSeQDs}$ NSs inset: normalized absorbance.

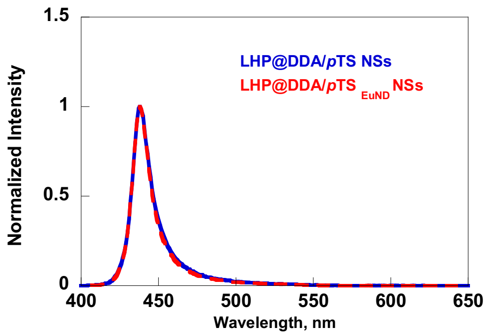

Figure S4. Comparison of the normalized emission spectra of LHP@DDA/*p*TS and LHP@DDA/*p*TS$_{EuND}$ NSs.

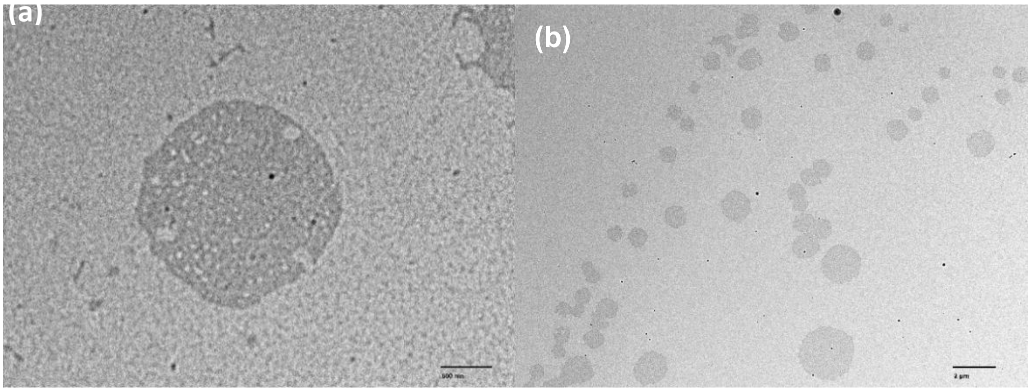

(c)
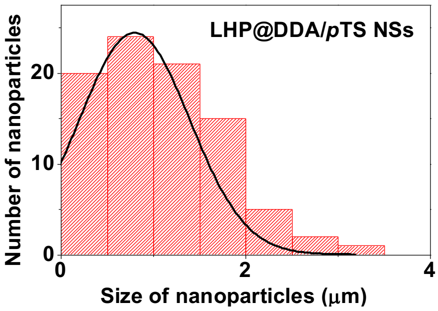

Figure S5. TEM images (scale bar (a) 500 nm and (b) 2 μm) and size distribution of LHP@DDA/*p*TS NSs (a-c).

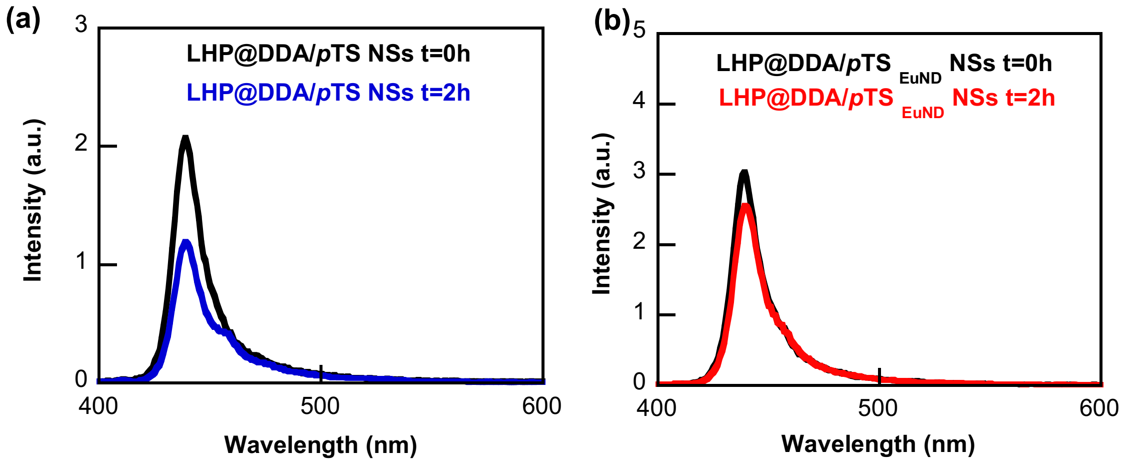

Figure S6. Photostability in air of (a) LHP@DDA/*p*TS NSs and (b) LHP@DDA/*p*TS_{EuND} NSs, λ_{ex} = 365 nm, 700V. $\lambda_{em} = 439$ nm.

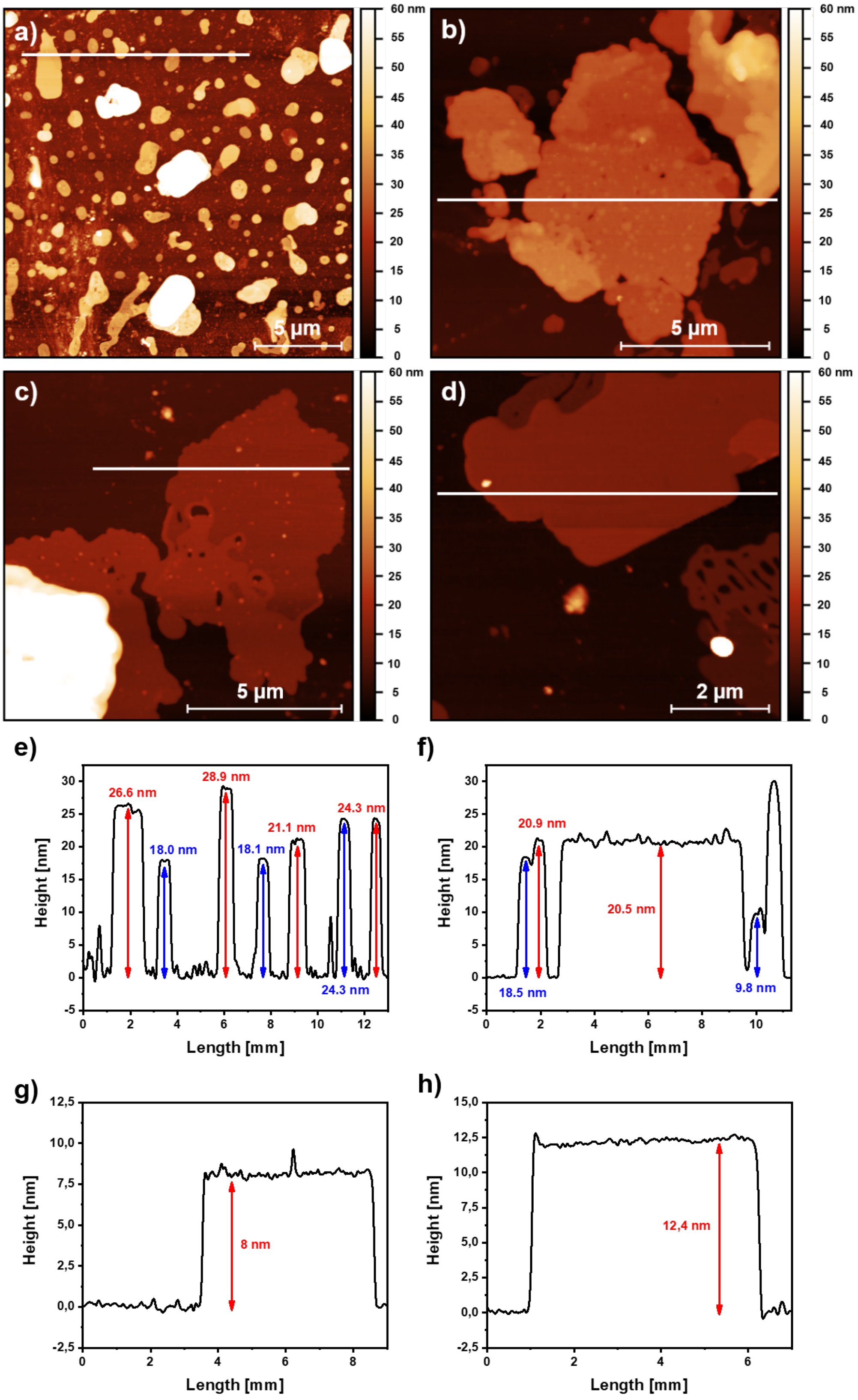

Figure S7. Topographic images obtained by AFM of different LHP@DDA/*p*TS$_{EuNDs}$ NSs isolated on a SiO2/Si substrate by drop-casting and their corresponding line profiles.

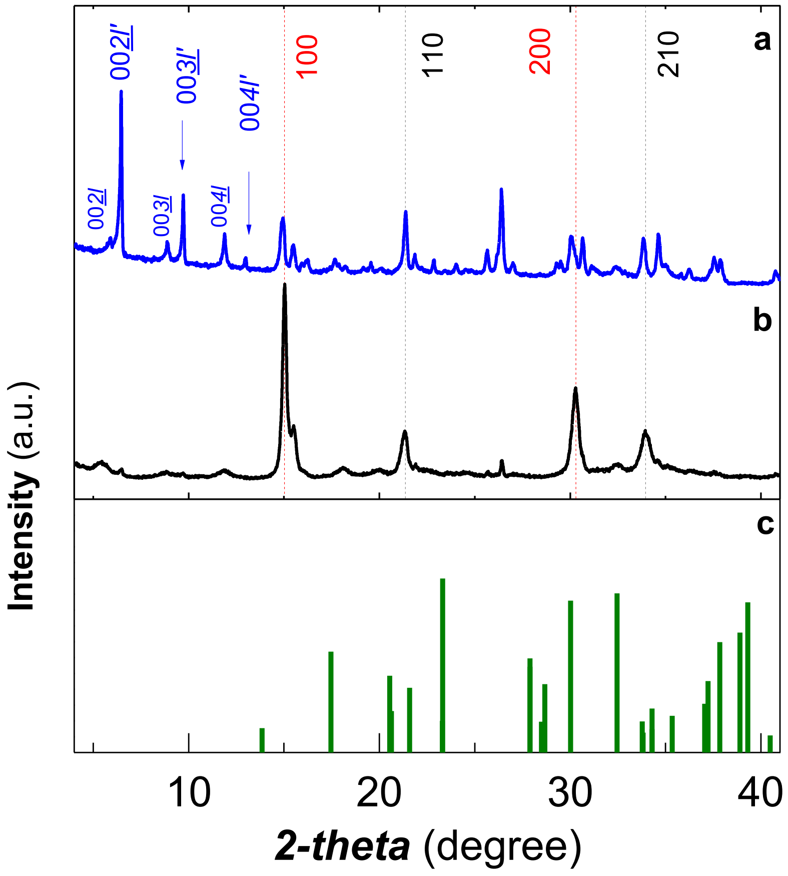

Figure S8. Powder X-ray diffraction (PXRD) of (a) LHPs recorded after the synthesis without any purification step (b) LHP@DDA/*p*TS_{EuND} NSs and (c) $PbBr_2$ XRD pattern.

XPS data were obtained for C_{1s}, O_{1s}, N_{1s}, Pb_{4f}, Br_{3d}, S_{2p} and Eu_{3d} elements in LHP@DDA/$_{EuND}$ NSs (Figure S8). The C_{1s} spectrum showed the typical signals of aliphatic and aromatic carbons, peaks at 284.5 eV, 285.6 and 286.7 eV, which can be ascribed to C-H, C-S and aromatic C=C, respectively. [2] In addition, the typical peaks of LHP for Pb_{4f} (at 138 eV and 143 eV) and Br_{3d} (at 67.9 eV and 68.9 eV) were also observed. The O_{1s} spectrum presented two peaks at 531.9 and 533.7 eV attributed to the O-O and O-S, respectively. The europium spectrum showed two peaks of Eu_{3d} at 1156 eV and 1165 eV, ascribed to Eu^{2+} and Eu^{3+}, respectively. The presence of Eu^{3+} was attributed to the oxidation of Eu^{2+} in the presence of water and oxygen, which could occur while registering the XPS spectrum, as it was previously reported for Eu^{2+} salts.[3] The possible formation of Eu_2O_3 was discarded due to the absence of the Eu-O peak at 530 eV in the O_{1s} spectrum.[4] The N_{1s} spectrum presented only a peak at 401.5 eV, ascribed to the NH_3^+ ammonium group of methylammonium and dodecylammonium, thereby confirming the protonation of DDA in the presence of *p*TS. Although the signal of S_{2p} in the 163-168 eV region was weak, it corroborated the presence of *p*TS ligand (spectrum not shown).[5] Data corroborated the presence of *p*TS and DDA ligands and the quantitative analysis displayed Br/Pb, DDA/Pb and Eu/Pb molar ratios of 2.8, 0.84 and 0.08, respectively and a total Eu atomic percentage of 0.2.

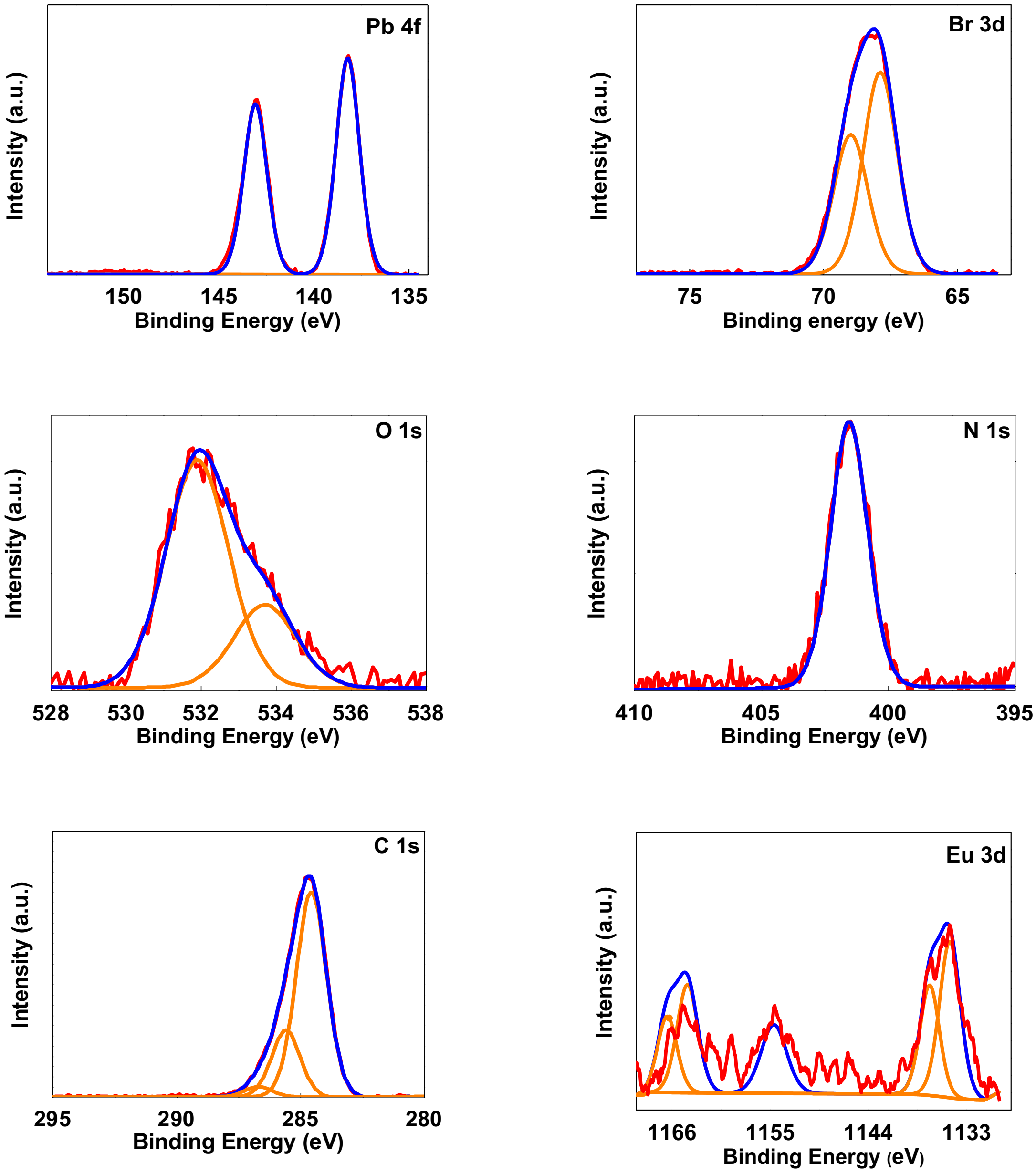

Figure S9. XPS spectra of Pb_{4f}, Br_{3d}, O_{1s}, N_{1s}, C_{1s}, and Eu_{3d} of the LHP@DDA/*p*TS$_{EuND}$ NSs.

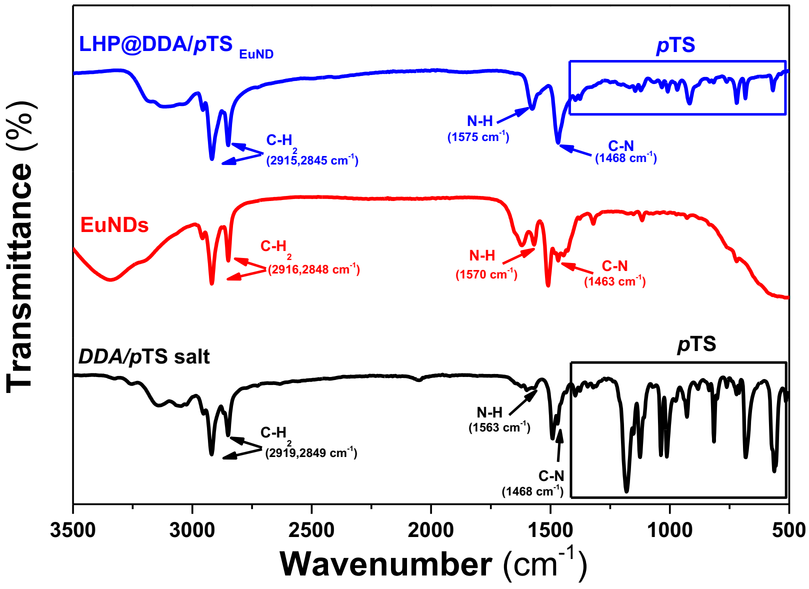

Figure S10. ATR-FTIR spectra of LHP@DDA/pTS$_{EuND}$ NSs, EuNDs, the DDA/*p*TS salt.

The ATR-FTIR spectrum of LHP@DDA/*p*TS$_{EuND}$ was registered and compared to those of EuNDs and the DDA/*p*TS salt (Figure S9). The main bands attributed to the DDA/*p*TS salt were observed : i) at 1468 cm^{-1} , ascribed to C-N stretching bands; ii) at 1575 cm^{-1}, attributed to to the N-H stretching bands (compared to 1563 cm^{-1} and 1570 cm^{-1} in the DDA/*p*TS salt and EuNDs, respectively); iii) at 2845 cm^{-1} and 2915 cm^{-1}, assigned to the symmetric and asymmetric C-H stretching vibrations (2849 cm^{-1} and 2919 cm^{-1} in the salt; 2848 cm^{-1} and 2916 cm^{-1} in the EuNDs);[6]24] and iv) in the 1370-600 cm^{-1} range, ascribed to symmetric and asymmetric stretching vibrations of sulfonate.[5]23][7] [7a]

LHP@DDA/*p*TS$_{EuNDs}$ NSs the ^{1}H-NMR of LHP@DDA/*p*TS NSs dissolved in deuterated dimethyl sulfoxide (*d*-DMSO), which reverted the perovskite material to its precursors, confirmed the presence of protonated DDA and *p*TS as the organic ligands with a DDA/ *p*TS molar ratio of 5.6 (Figure S10-12). ^{1}H NMR (300 MHz, DMSO deuterated) ppm: 0.85 (m,3H), 1.26 (s,20H), 1.5 (m, 2H), 2.75 (t, 2H), 7.14 (d, *J*=4.8, 2H), 7.53 (d, *J*=4.8, 2H)

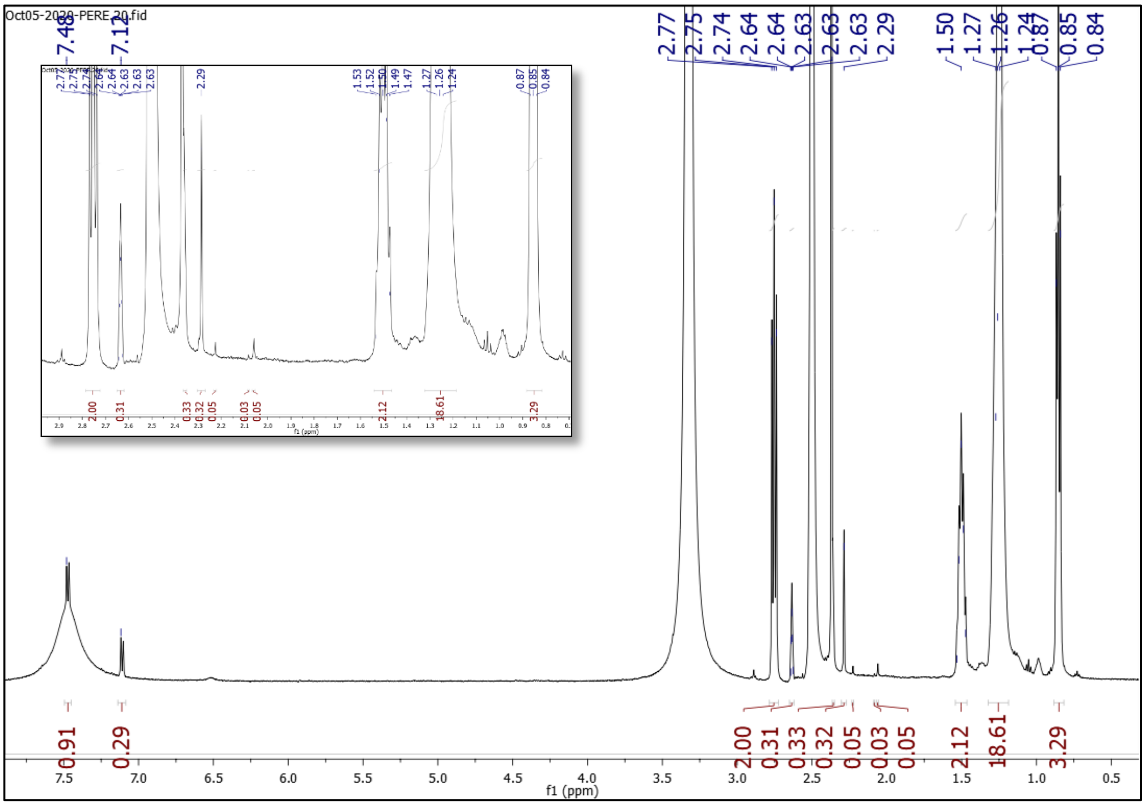

Figure S11. ^{1}H NMR of LHP@DDA/*p*TS$_{EuNDs}$ NSs in *d*-DMSO.

DDA

^{1}H NMR (300 MHz, *d*-DMSO) ppm: 0.86 (m,3H), 1.25 (s,20H), 1.51 (m, 2H), 2.76 (t,2H)

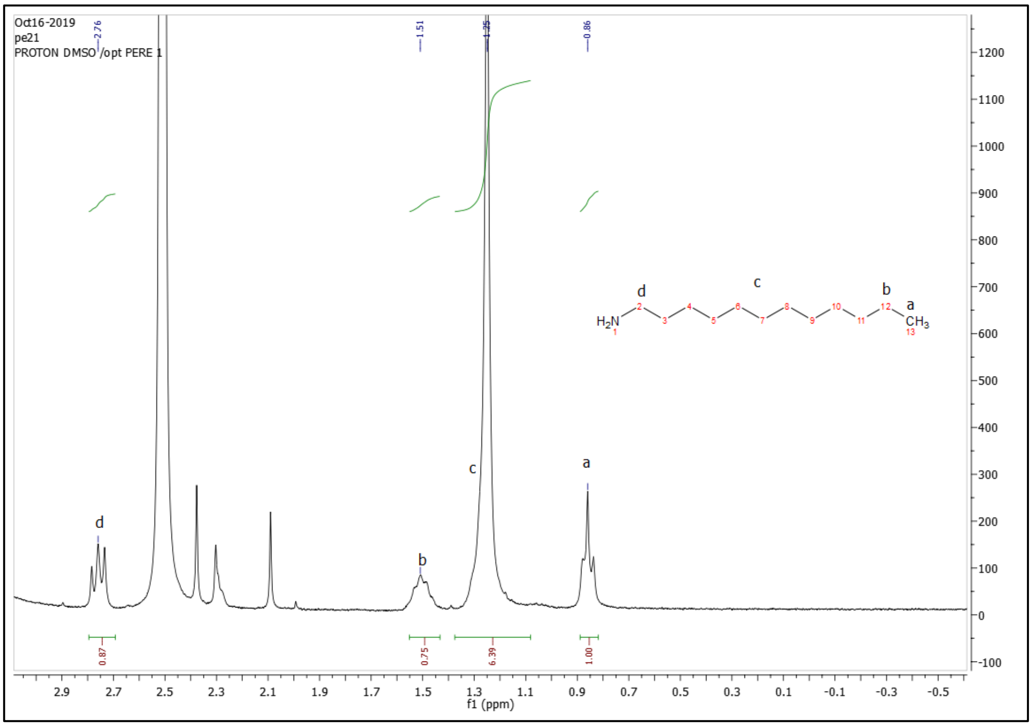

Figure S12. ^{1}H-NMR of DDA in *d*-DMSO.

***p*-toluensulfonic acid**

^{1}H NMR (300 MHz, *d*-DMSO) ppm: 2.30 (s,3H), 7.14 (d, *J*=7.8, 2H), 7.53 (d, *J*=7.8, 2H)

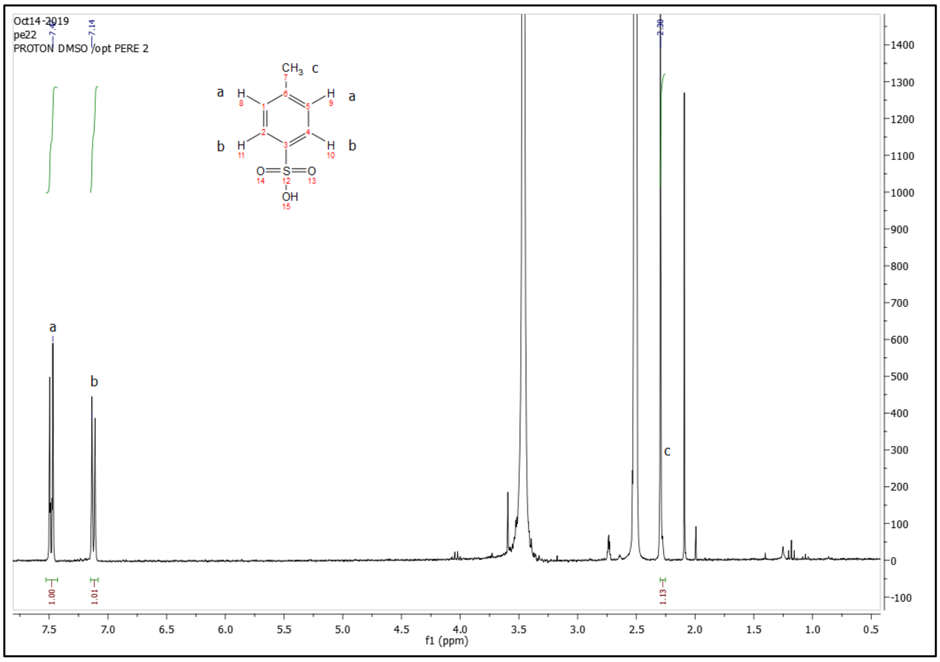

Figure S13. ^{1}H-NMR of *p*TS in *d*-DMSO.

Thermogravimetry Analysis coupled with Gas Chromatography and Mass Spectrometry

Thermogravimetric analysis coupled with gas chromatography and mass spectrometry (TG–GC–MS) is a powerful technique to unveil the thermal stability of hybrid materials, the chemical interaction between components, as well as the thermal decomposition products and pathways.[8] In order to rationalize the composition of LHP@DDA/*p*TS$_{EuND}$ NSs, the thermal behavior of the ligands (*p*-toluenesulfonic acid and dodecylamine), the europium dots (EuNDs), and the LHP@DDA/*p*TS$_{EuND}$ NSs have been analyzed separately. As a first step we decided to analyze the thermal behavior and the decomposition products of the ligands: *p*TS and DDA (see supporting information for details, Figure S13-S23).

***p*-Toluenesulfonic acid.** Figure S13 depicts the TG profile for thermal decomposition of *p*TS sample. By analyzing the first derivative of the TG signal (DTG), at least two well-defined steps can be noticeable. While the first one takes place at 85 °C, the second one occurs at 235 °C, exhibiting a small shoulder around 285 °C. The evolved gaseous fragments from each decomposition step were injected in a gas chromatography column at 40 °C (to enhance the retention time of the smallest molecules) keeping in mind to identify the chemical processes that take place. The first step that exhibits a mass loss of around 10% can be assigned to the loss of water molecules (first injection), as expected according to the formula: $CH_3C_6H_4SO_3H{\cdot}H_2O$, as shown in Figure S14. The second step exhibits a mass loos of *ca*. 90%, where the lack of solid residues at the end of the measurement suggested either the total decomposition or sublimation of the organic molecule. The elugrams recorded from the second and the third injections at 236 and 283 °C (chromatography column at 40 °C), depict characteristic fragments of *p*TS molecule, such as sulfur dioxide (SO_2) and toluene (Figure S14).

Additionally, the decomposition was repeated by injecting the evolved gaseous fragments at 285 °C and heating the chromatography column to 250 °C (to observe the largest fragments/molecules). The elugram from Figure S15 shows the presence of different structural isomers from the di-substituted diaryl sulfone. These fragments suggest the decomposition of *p*TS molecules during the thermal heating, providing characteristic signals for its identification. Table S2 compiles the assignment of the signals by employing NIST MS Search 2.3 software.

Table S2. Identified molecules after injecting at 283 °C the evolved gases of the thermal decomposition under inert atmosphere of *p*TS.

#	Molecule	Match	R. Match	Prob	Structure
(1)	Phtalimide	852	852	67	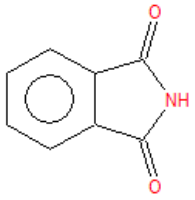
(2)	Isoquinaldamide	615	620	24	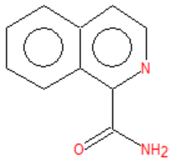
(3)	1-methyl-4(phenylsilfonyl)-benzene	677	758	94	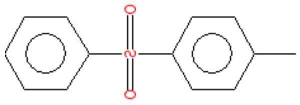
(4)	1,1’-sulfonylbis[2-methyl-benzene	786	816	93	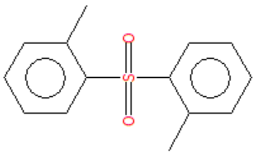
(5)	di-p-tolyl sulfone	793	874	91	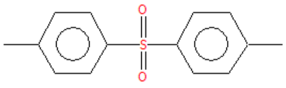
(6)		903	948	97	
(7)	Benzo[1,2-c:4,5-c']dipyrrole-1,3,5,7(2H,6H)-tetrone	244	36	15	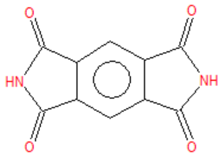

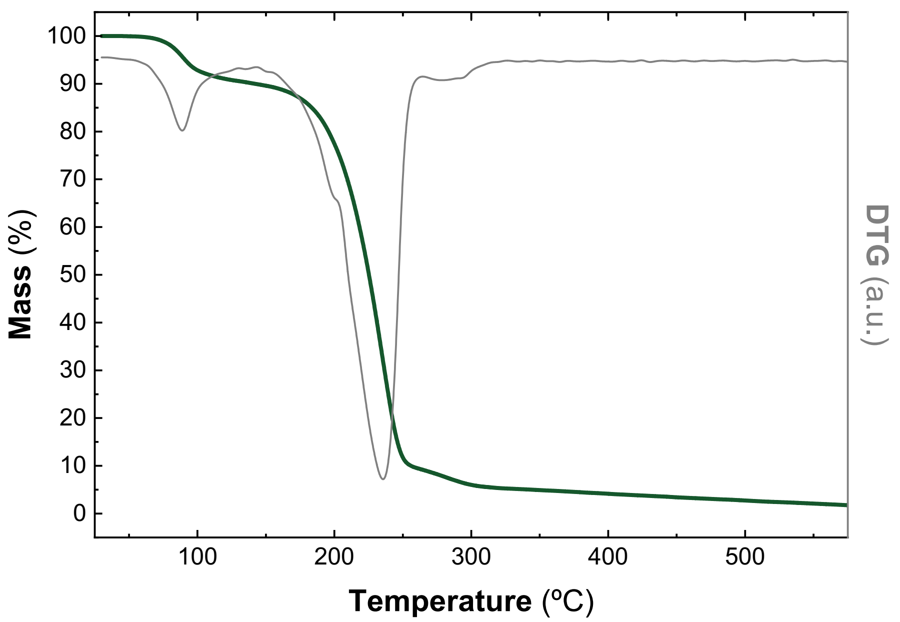

Figure S14. TG profile for *p*TS recorded under inert conditions (He: 20 mL/min) at 10 °C/min. Grey line represents the first derivative (DTG).

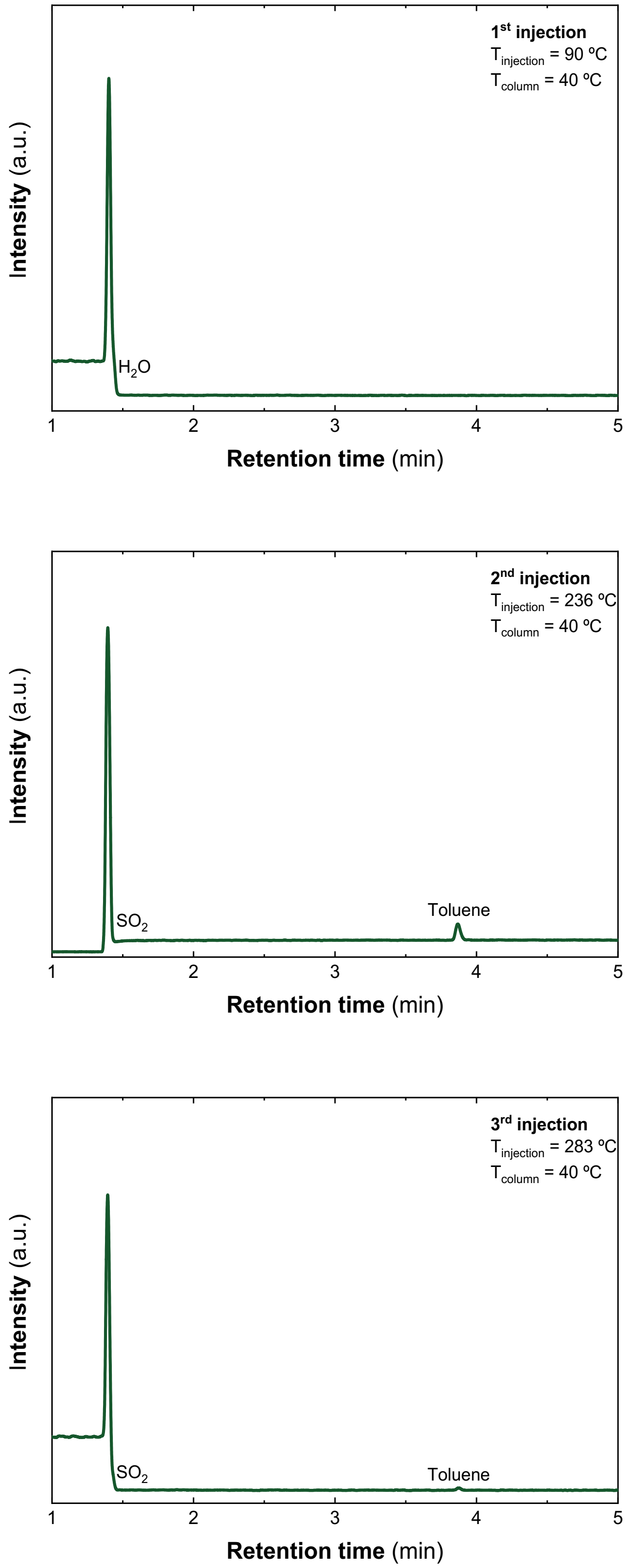

Figure S15. Chromatogram corresponding to the injection recorded at 90 °C (upper panel), 236 °C (middle panel) and 283 °C (lower panel) for sample *p*TS. Chromatography column temperature: 40 °C.

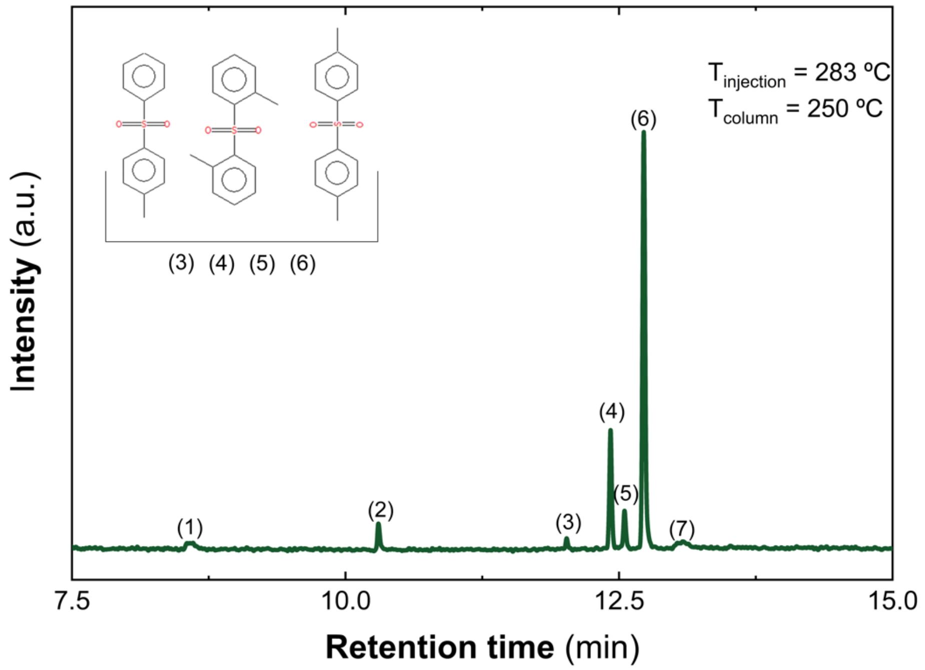

Figure S16. Chromatogram corresponding to the injection recorded at 283 °C for sample *p*TS. Chromatography column temperature: 40 °C. Signals were analyzed by employing NIST MS Search 2.3 software, and compiled in Table S2.

Dodecylamine (DDA). Figure S16 depicts the TG profile for thermal decomposition of the DDA sample exhibiting only one step. A broad decomposition step in the range from 95 to 145 °C can be observed with non-solid residues at the end of the measurement. This suggests either the total decomposition or sublimation of the organic molecule. In this case only one injection was performed at 110 °C. When the gas chromatography column was set at 40 °C no signal was observed even after 90 min of elution. Hence, the injection was repeated with the chromatography column heated at 250 °C. Figure S17 depicts the elugram where more than 10 signals can be noticeable. Each signal was assigned by employing NIST MS Search 2.3 software and compiled in Table S3. After the analysis of the signals, undecanitrile (labeled as 12 in Figure S17) arises as the most characteristic fragment to follow to thermal decomposition of DDA ligand.

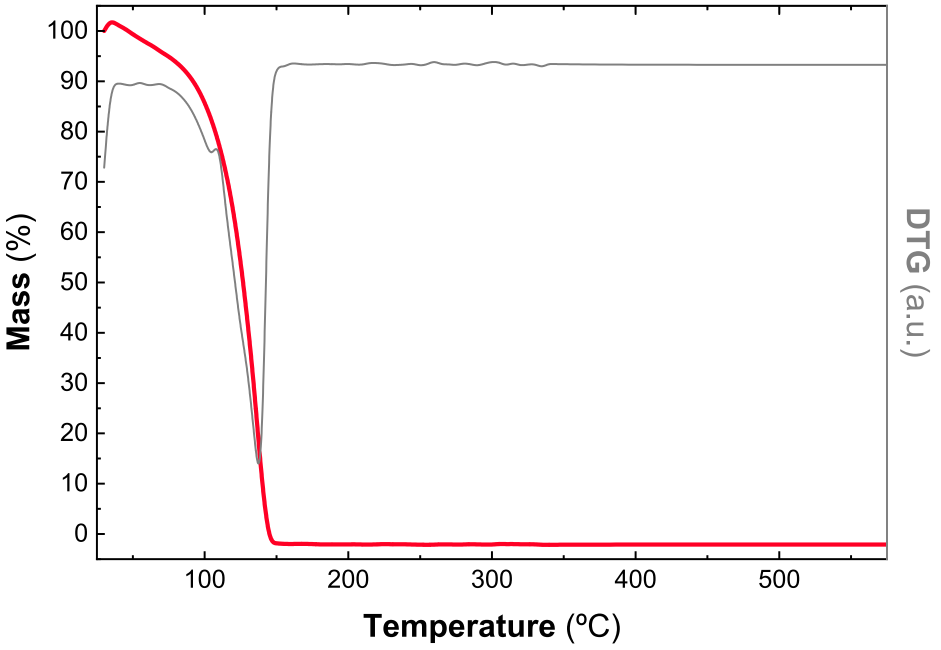

Figure S17. TG profile for sample DDA recorded under inert conditions (He: 20 mL/min) at 10 °C/min. Grey line represents the first derivative (DTG).

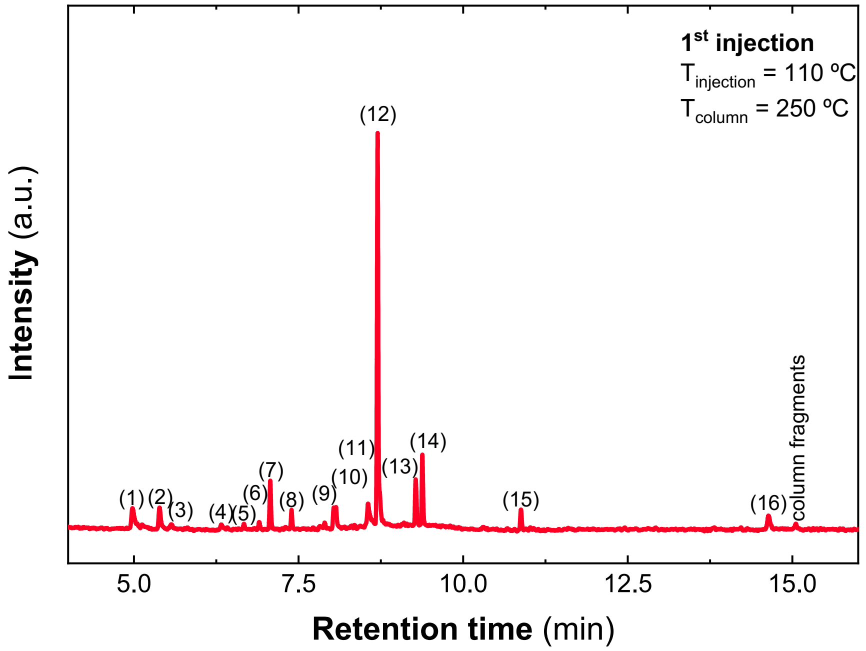

Figure S18. Chromatogram corresponding to the third injection recorded at 110 °C for sample DDA. GC column temperature: 250 °C. Signals were analyzed by employing NIST MS Search 2.3 software and compiled in Table S3.

Table S3. Identified molecules after injecting at 110 °C the evolved gases of the thermal decomposition under inert atmosphere of DDA.

#	Molecule	Match	R. Match	Prob	Structure
(1)	4-tetradecene (Z)	805	818	4.4	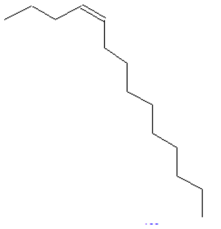
(2)	Decanal	803	817	13.6	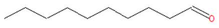
(3)	1-tetradecene	726	779	3.11	
(4)	2-(Phenylacetamido)propionic acid	638	669	31.7	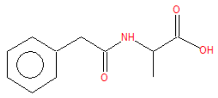
(5)	2-butyltetrahydrofurano	596	783	30.4	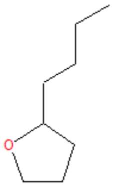
(6)	2-N-octylfuran	627	743	43.8	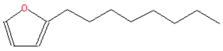
(7)	undecanal	783	890	27.3	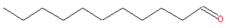
(8)	2-dodecenal	647	765	4.82	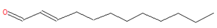
(9)	2-pentyl-pyridine	660	828	18.6	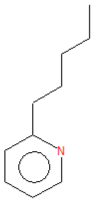
(10)	undecanal	528	774	27.2	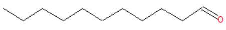
(11)	2-[1-(4-Nitrobenzoylhydrazono)ethyl]benzoic acid	457	656	10.9	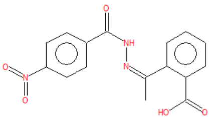
(12)	undecanitrile	833	912	40.1	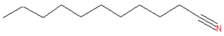
(13)	1-isocyanato-octadecene	609	805	19.2	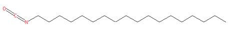
(14)	Undecanitrile	649	862	15.1	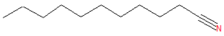
(15)	Hexadecyl formamide	673	835	62.6	

(16)	2-Ethyl-2-phenyl-1,3-benzodioxole	685	880	39.4	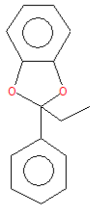

EuNDs precursor (EuNDs). Once we have characterized the thermal behavior of the ligands, we carried out an analogous analysis over the precursor EuNDs. Figure S18 depicts the thermal decomposition of EuNDs, where more than 50% of mass is retained, attributable to the presence of the inorganic phase. The analysis of DTG reveals different steps, however the most prominent takes place at 260 °C, where the evolved gaseous fragments were injected. As in the case of DDA ligand, no signals were found when the gas chromatography column was set at 40 °C, even after 90 min. Therefore, the injection was repeated with the chromatography column heated at 250 °C, the elugram reveals the presence of 6 signals (Figure S19). Interestingly, the two most significant signals are attributed to dodecylamine and undecanitrile, thus confirming the presence of DDA in the precursor. Additionally, the other signals were assigned by employing NIST MS Search 2.3 software and compiled in Table S4.

Table S4. Identified molecules after injecting at 260 °C the evolved gases of the thermal decomposition under inert atmosphere of EuNDs.

#	Molecule	Match	R. Match	Prob	Structure
(1)	1-dodecanamine	679	846	15	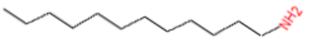
(2)	Undecanetrile	817	884	44	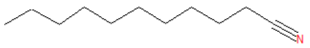
(3)	1-isocyanato-dodecane	711	764	35	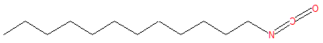
(4)	1.2-benzenedicarbonitrile	570	691	35	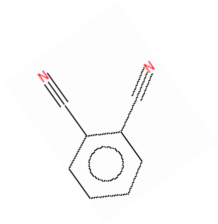
(5)	Benzo[1,2-c:4,5-c']dipyrrole-1,3,5,7(2H,6H)-tetrone	741	828	85	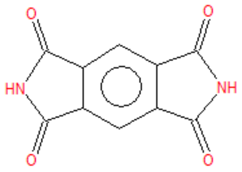

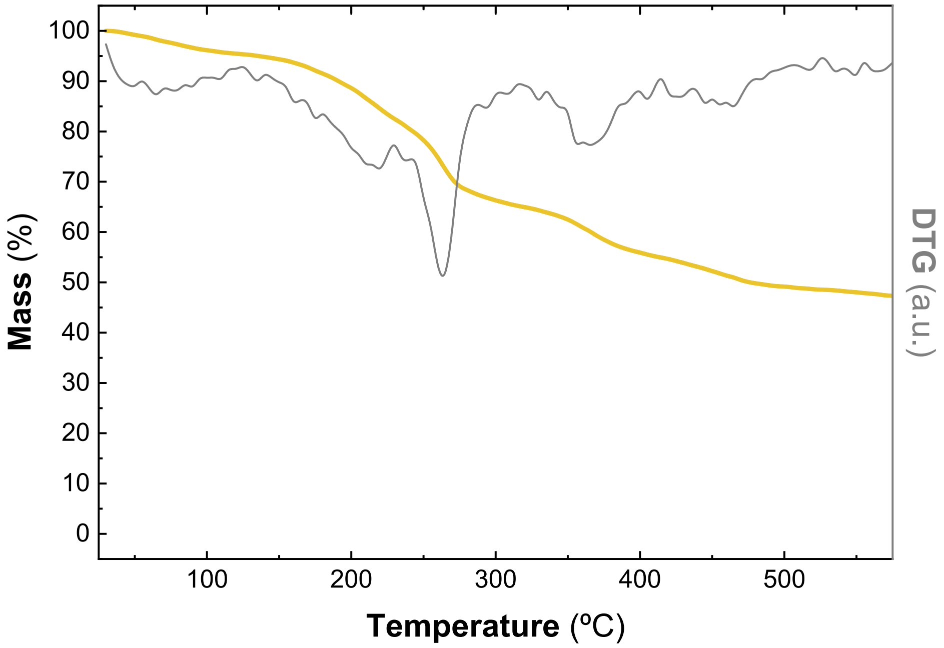

.

Figure S19. TG profile for sample EuNDs recorded under inert conditions (He: 20 mL/min) at 10 °C/min. Grey line represents the first derivative (DTG).

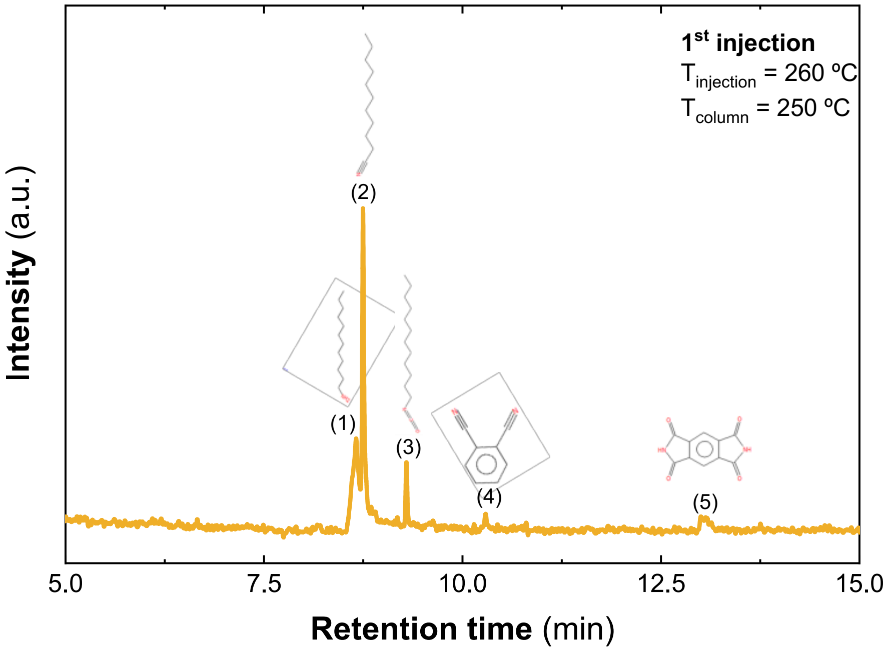

Figure S20. Chromatogram corresponding to the first injection recorded at 260 °C for sample EuNDs. GC column temperature : 250 °C. Signals were analyzed by employing NIST MS Search 2.3 software and compiled in Table S4.

LHP@DDA/*p*TS$_{EuND}$ hybrid. Once we have characterized the ligands and the precursor, we performed the thermal decomposition of our hybrid LHP@DDA/*p*TS$_{EuND}$. Figure S20 depicts the TG profile for thermal decomposition of LHP@DDA/*p*TS$_{EuND}$ hybrid, where two well-defined steps at 285 and 500 °C can be observed. In order to evaluate to smallest molecules, different injections were performed in a gas chromatography column heated at 40 °C. In both cases, 285 and 500 °C, the signals can be assigned to sulfur dioxide confirming the presence of *p*TS in the hybrid and its decomposition along the heating process (Figure S21). In this case, after 90 minutes with the GC column at 40 °C, the temperature was increased to 250 °C in order to detach the largest molecules. Figure S22 depicts the elugram after both injections. Interestingly, the presence of the characteristic molecules observed for *p*TS and DDA decomposition, such as di-substituted diaryl sulfone and undecanitrile, are observable. These results confirm the presence of both ligands in our LHP@DDA/pTS$_{EuND}$ hybrid. The analysis of all the signals employing NIST MS Search 2.3 software is compiled in Table S5.

Table S5. Identified molecules after collecting with the column at 40 °C both injections at 285 and 500°C of the evolved gases of the thermal decomposition under inert atmosphere of LHP@DDA/*p*TS$_{EuND}$ hybrid.

#	Molecule	Match	R. Match	Prob	Structure
(1)	Phthalimide	669	674	15	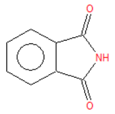
(2)	Undecanitrile	827	866	34	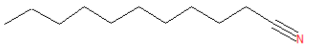
(3)	1-isocyanato-dodecane	834	860	65	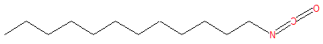
(4)	1.2-benzenedicarbonitrile	621	643	13	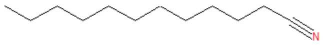
(5)	Isoquinaldamide	725	736	69	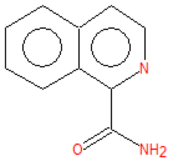
(6)	1-methyl-4(phenylsulfonyl)-benzene	626	720	93	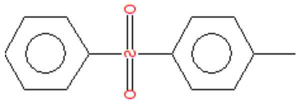
(7)	1,1'-sulfonylbis[2-methyl-benzene]	732	768	82	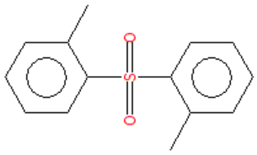
(8)	di-*p*-tolyl sulfone	916	951	98	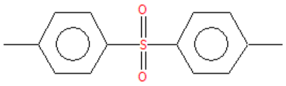
(9)	Benzo[1,2-c:4,5-c']dipyrrole-1,3,5,7(2H,6H)-tetrone	808	879	86	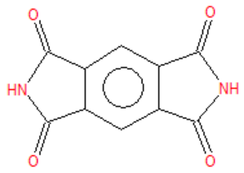

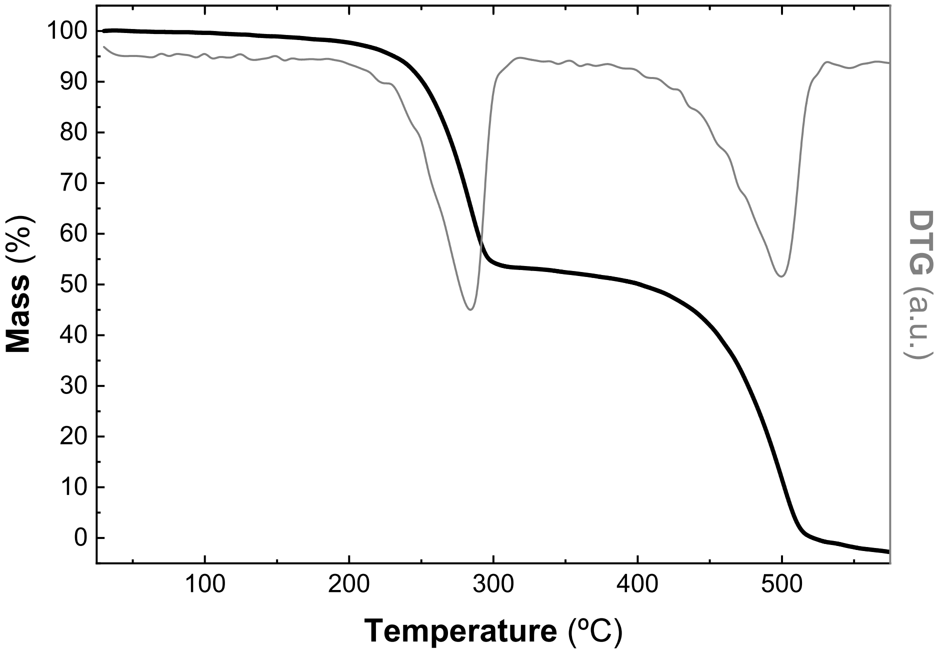

Figure S21. TG profile for sample LHP@DDA/*p*TS$_{EuND}$ NSs hybrid recorded under inert conditions (He: 20 mL/min) at 10 °C/min.

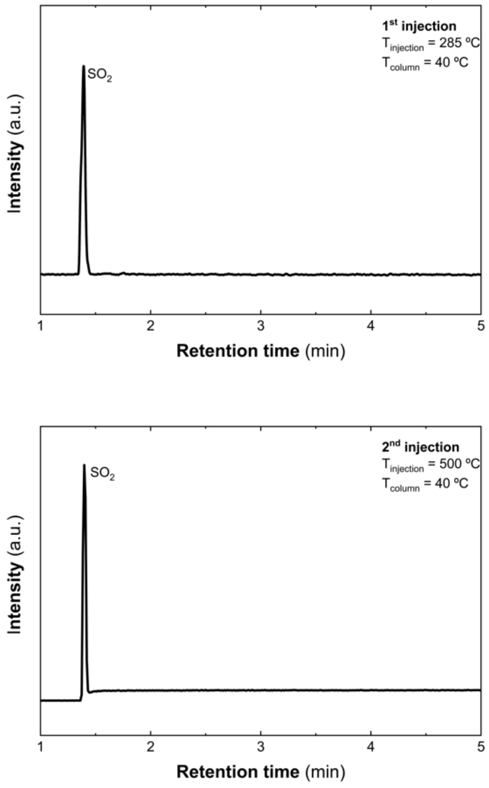

Figure S22. Chromatogram corresponding to the injections recorded at 285 °C (upper panel) and 500 (lower panel) for LHP@DDA/*p*TS_{EuND} NSs hybrid. GC column temperature: 40 °C.

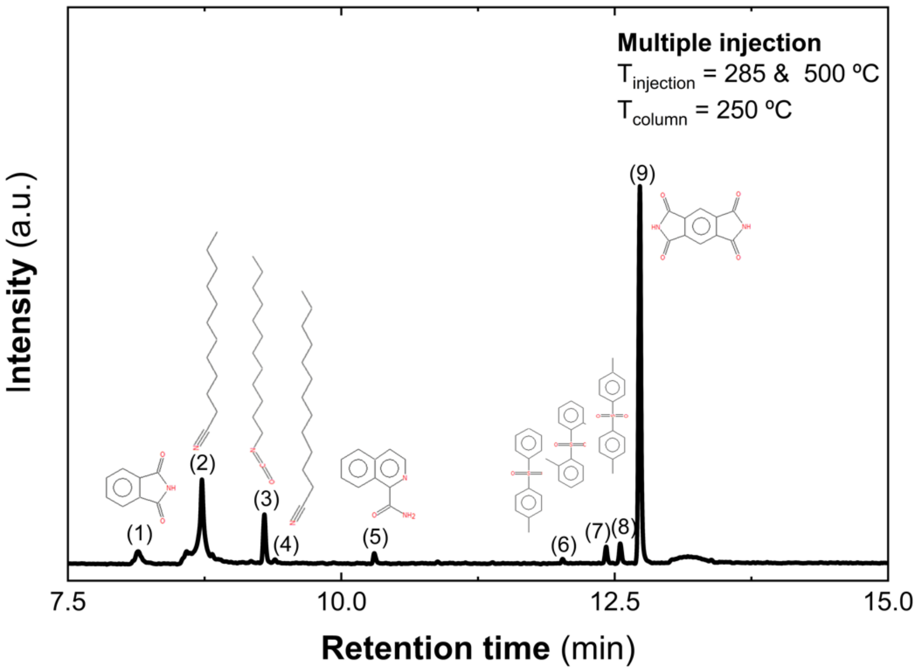

Figure S23. Chromatogram corresponding to a multiple injection process recorded at 285 and 500 °C for sample LHP@DDA/*p*TS$_{EuND}$ NSs. GC column temperature: 250 °C. Signals were analysed by employing NIST MS Search 2.3 software and compiled in Table S5.

Finally, in order to precisely assign the loss of the ligands during the thermal decomposition, another TG–GC–MS experiment was carried out by injecting the evolved gaseous fragment in the column at 250 °C, exclusively. Figure S23 depicts the elugram of LHP@DDA/pTS$_{EuND}$ NSs hybrid decomposition after the injection performed in the first step at 285 °C. Interestingly, the major signal is attributed to the presence undecanitrile, a fingerprint of the decomposition of DDA molecule, as it was also observed in the case of the precursor (Figure S19). Additionally, other characteristic molecules, such as 1-isocyanato-dodecane and 1.2-benzenedicarbonitrile, were observed. Table S6 compiles the assignment of the signals by employing NIST MS Search 2.3 software.

Table S6. Identified molecules after injecting at 285 °C the evolved gases of the thermal decomposition under inert atmosphere of LHP@DDA/*p*TS$_{EuND}$ hybrid.

#	Molecule	Match	R. Match	Prob	Structure
(1)	Phthalimide	648	734	21	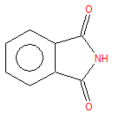
(2)	Undecanitrile	841	885	44	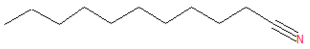
(3)	1-isocyanato-dodecane	551	623	11	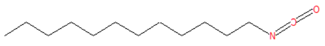
(4)	1.2-benzenedicarbonitrile	671	675	62	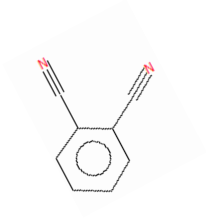
(5)	Benzo[1,2-c:4,5-c']dipyrrole-1,3,5,7(2H,6H)-tetrone	799	865	88	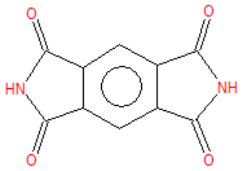

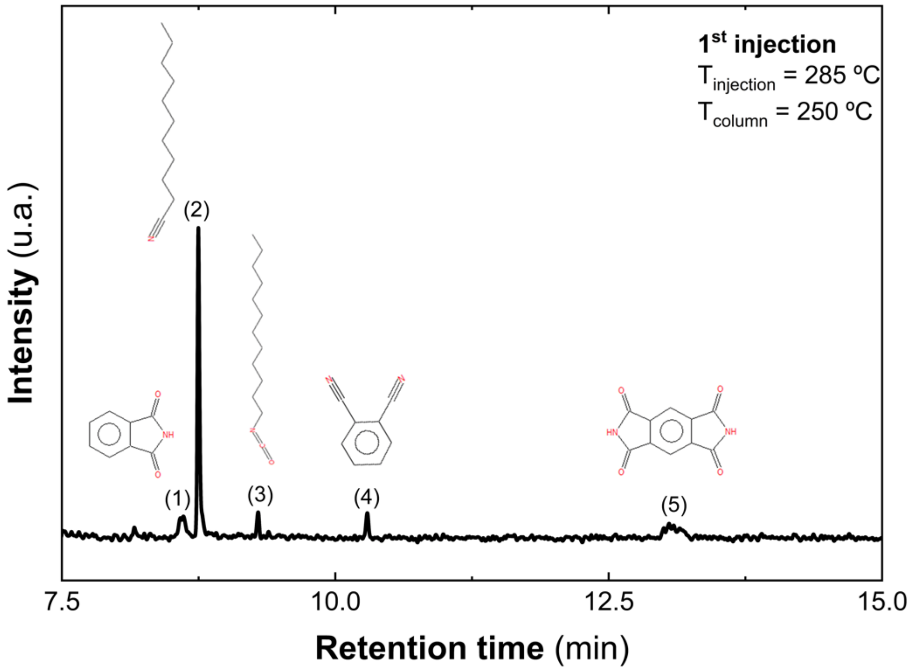

Figure S24. Chromatogram corresponding to the first injection recorded at 285 °C for sample LHP@DDA/*p*TS_{EuND} NSs hybrid. GC column temperature: 250 °C. Signals were analyzed by employing NIST MS Search 2.3 software and compiled in Table 5.

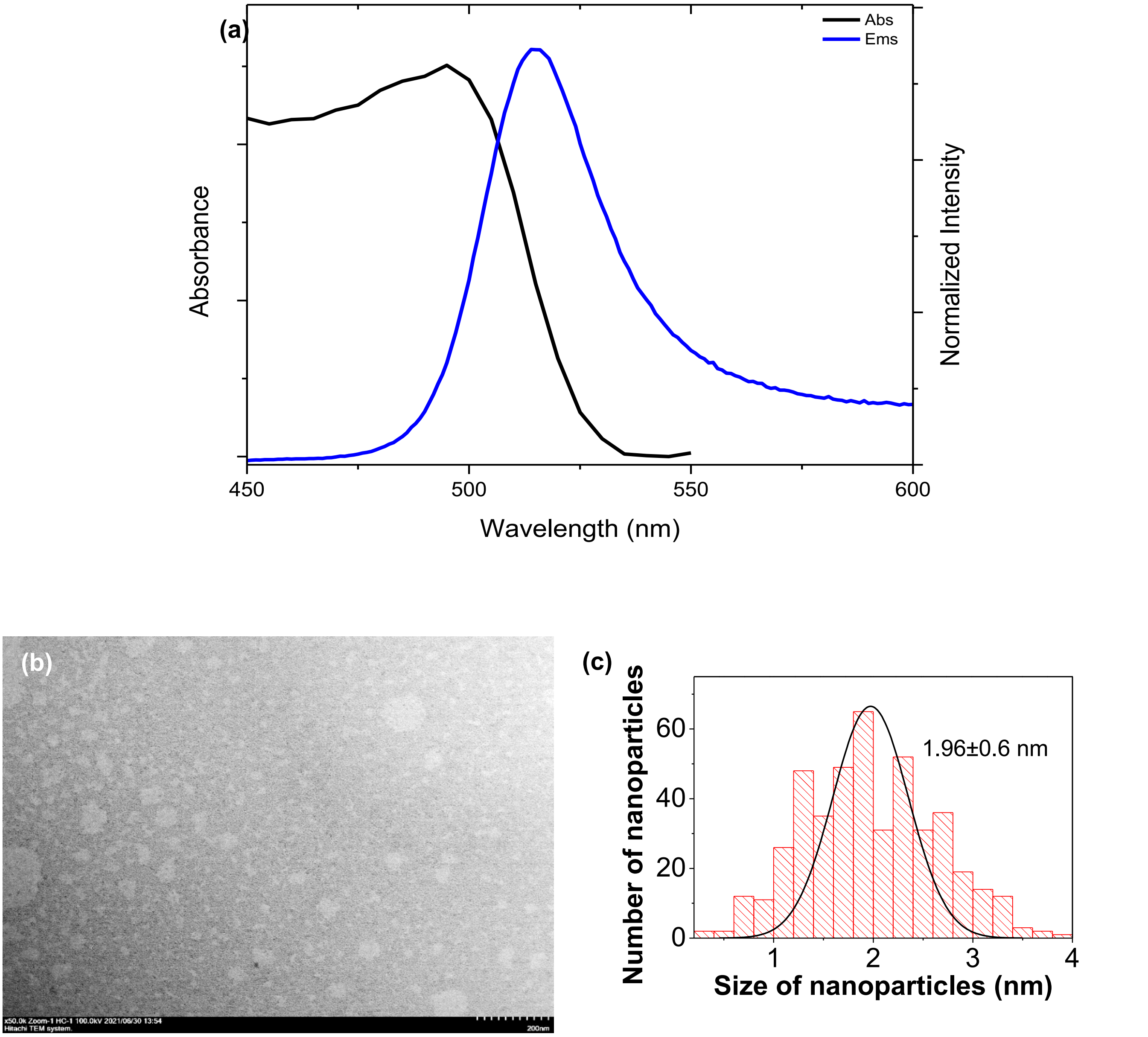

Figure S25. (a) Absorption and emission spectra of QDs CdSe, in toluene (λ_{ex} = 400 nm). (b) Transmission electron microscope (TEM) image of QDs CdSe (scale bar: 200nm). (c) Size distribution of the QDs CdSe.

The size of the CdSe QDs were estimated by absorption, emission and TEM microscopy.

- **Absorption[9]**

***D*(*nm*) =** $59.60816 - 0.54736\lambda + 1.8873\text{x}10^{-3}\lambda^{2} - 2.85743\text{x}10^{-6}\lambda^{3} + 1.62974\text{x}10^{-9}\lambda^{4}$ = **2.43 nm**

- **Emission[9]**

***D*(*nm*) =** $0.0566e^{0.0071\lambda}$ = **2.19 nm**

- **TEM images** (histogram, Figure 24c)

***D*(*nm*) =** 1.96 ± 0.6 nm

References

[1] I. Rosa-Pardo, S. Pocoví-Martínez, R. Arenal, R. E. Galian, J. Pérez-Prieto, *Nanoscale* **2019**, *11*, 18065-18070.

[2] C. A. d. S. Moura, G. K. Belmonte, P. G. Reddy, K. E. Gonsalves, D. E. Weibel, *RSC Advances* **2018**, *8*, 15029-15029.

[3] D. Kim, Y.-H. Jin, K.-W. Jeon, S. Kim, S.-J. Kim, O. H. Han, D.-K. Seo, J.-C. Park, *RSC Advances* **2015**, *5*, 74790-74801.

[4] N. Nasani, D. Ramasamy, I. Antunes, J. Perez, D. P. Fagg, *Electrochimica Acta* **2015**, *154*, 387-396.

[5] S. Kang, G. Zhang, X. Yang, H. Yin, X. Fu, J. Liao, J. Tu, X. Huang, F. G. F. Qin, Y. Xu, *Energy & Fuels* **2017**, *31*, 2847-2854.

[6] T. T. Nguyen, P. Bandyopadhyay, X. Li, N. H. Kim, J. H. Lee, *Journal of Membrane Science* **2017**, *540*, 108-119.

[7] a) U. U. Ozkose, C. Altinkok, O. Yilmaz, O. Alpturk, M. A. Tasdelen, *European Polymer Journal* **2017**, *88*, 586-593; b) A. G. Abd El Raouf Y. M, and Elsharawy T, *Life Science Journal* **2015**, *12*, 105-112.

[8] a) V. Oestreicher, D. Hunt, R. Torres-Cavanillas, G. Abellán, D. A. Scherlis, M. Jobbágy, *Inorganic Chemistry* **2019**, *58*, 9414-9424; b) E. Conterosito, L. Palin, D. Antonioli, D. Viterbo, E. Mugnaioli , U. Kolb, L. Perioli, M. Milanesio, V. Gianotti, *Chemistry – A European Journal* **2015**, *21*, 14975-14986; c) J. A. Carrasco, J. Romero, M. Varela, F. Hauke, G. Abellán, A. Hirsch, E. Coronado, *Inorganic Chemistry Frontiers* **2016**, *3*, 478-487; d) G. Abellán, M. Schirowski, K. F. Edelthalhammer, M. Fickert, K. Werbach, H. Peterlik, F. Hauke, A. Hirsch, *Journal of the American Chemical Society* **2017**, *139*, 5175-5182; e) J. Romero, M. Varela, M. Assebban, V. Oestreicher, A. Guedeja-Marrón, J. L. Jordá, G. Abellán, E. Coronado, *Chemical Science* **2020**, *11*, 7626-7633; f) V. Oestreicher, G. Abellán, E. Coronado, *Physica status solidi RRL* **2020**, *14*, 2000380.

[9] M. L. Landry, T. E. Morrell, T. K. Karagounis, C.-H. Hsia, C.-Y. Wang, *Journal of Chemical Education* **2014**, *91*, 274-279.
